# Supplementary material for: Pharmacogenomics of poor drug metabolism in greyhounds: Canine P450 oxidoreductase genetic variation, breed heterogeneity, and functional characterization
Source: PLoS One. 2024 Feb 1;19(2):e0297191. doi: 10.1371/journal.pone.0297191 (PMC10833530; doi:10.1371/journal.pone.0297191)

S1 Fig. *POR* haplotype frequency heterogeneity across dog breeds. *POR*-H2, -H3, and H4 haplotypes were determined by genotyping DNA samples from 21 sighthound breeds, 47 other breeds, and mixed-breed dogs. At least 10 dogs were sampled per breed. Breeds were designated by the dog's owner. Greyhounds were divided into two breed sub-groups based on whether their owner identified them as a dog registered with the National Greyhound Association (NGA*) bred for racing or a dog registered with the American Kennel Club (AKC**) bred for other purposes. The numbers of individual dogs that were sampled are shown next to each dog breed.

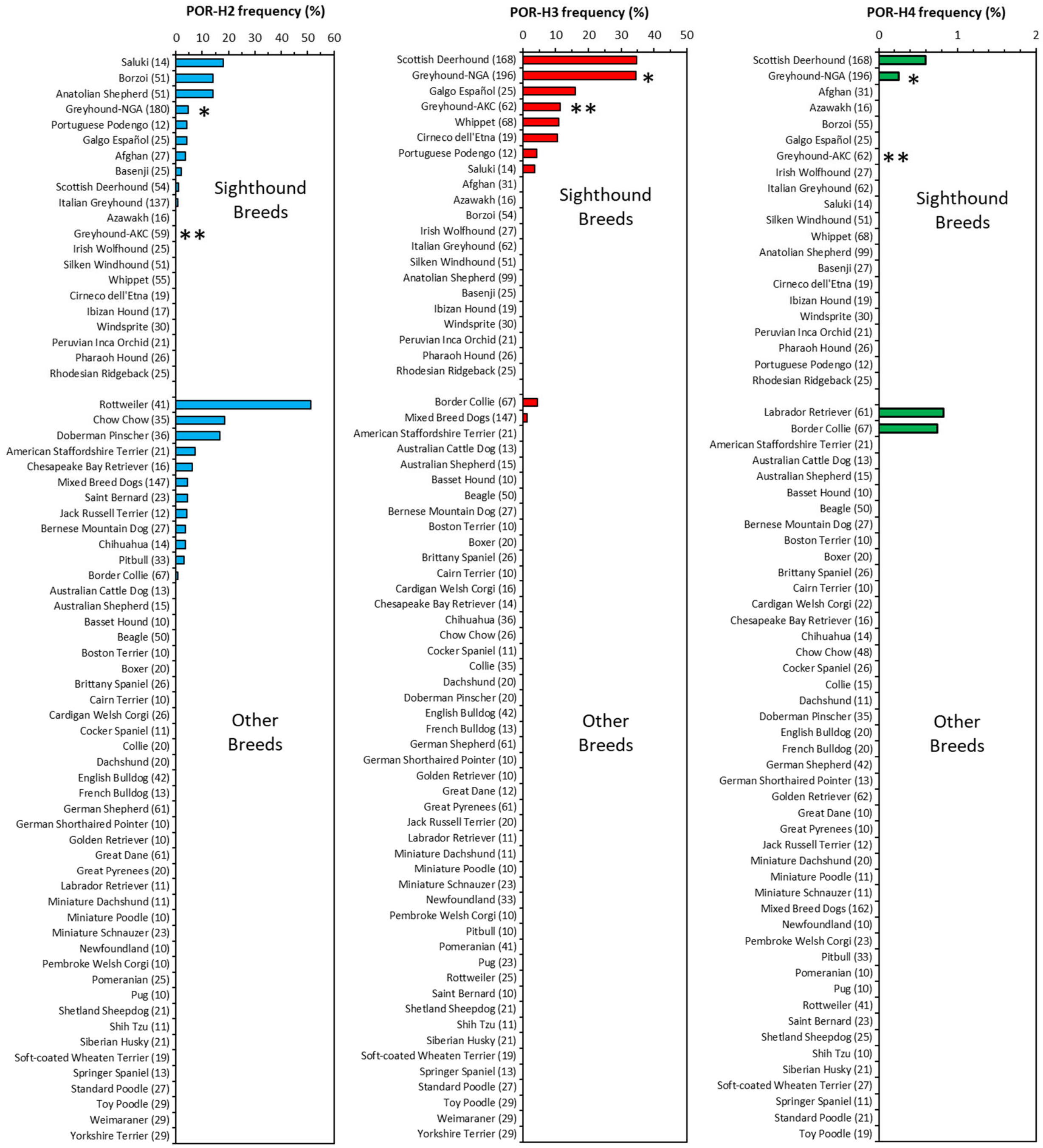

Supplement: S1 Fig — (PDF) [file pone.0297191.s001.pdf]
