# Supplementary material for: Pharmacogenomics of poor drug metabolism in greyhounds: Canine P450 oxidoreductase genetic variation, breed heterogeneity, and functional characterization
Source: PLoS One. 2024 Feb 1;19(2):e0297191. doi: 10.1371/journal.pone.0297191 (PMC10833530; doi:10.1371/journal.pone.0297191)

S2 Fig. Full-length immunoblots of cropped blots displayed in Figs. 5A, 5B, and 5C. Single bands of the expected protein size were noted for POR (77 kDa) and CYP2B11 (54 kDa). In the CYP2D15 blot, in addition to the expected band at 54 kDa, a faster migrating band (about 10 kDa smaller) was observed. This band was also found in uninfected Sf9 cells, but absent from pDLMs suggesting that it is a crossreactive background protein expressed in Sf9 cells. The 54 kDa CYP2D15 band was used for quantitation purposes. See legends to Figures S3-5 for details regarding how the blots were generated.

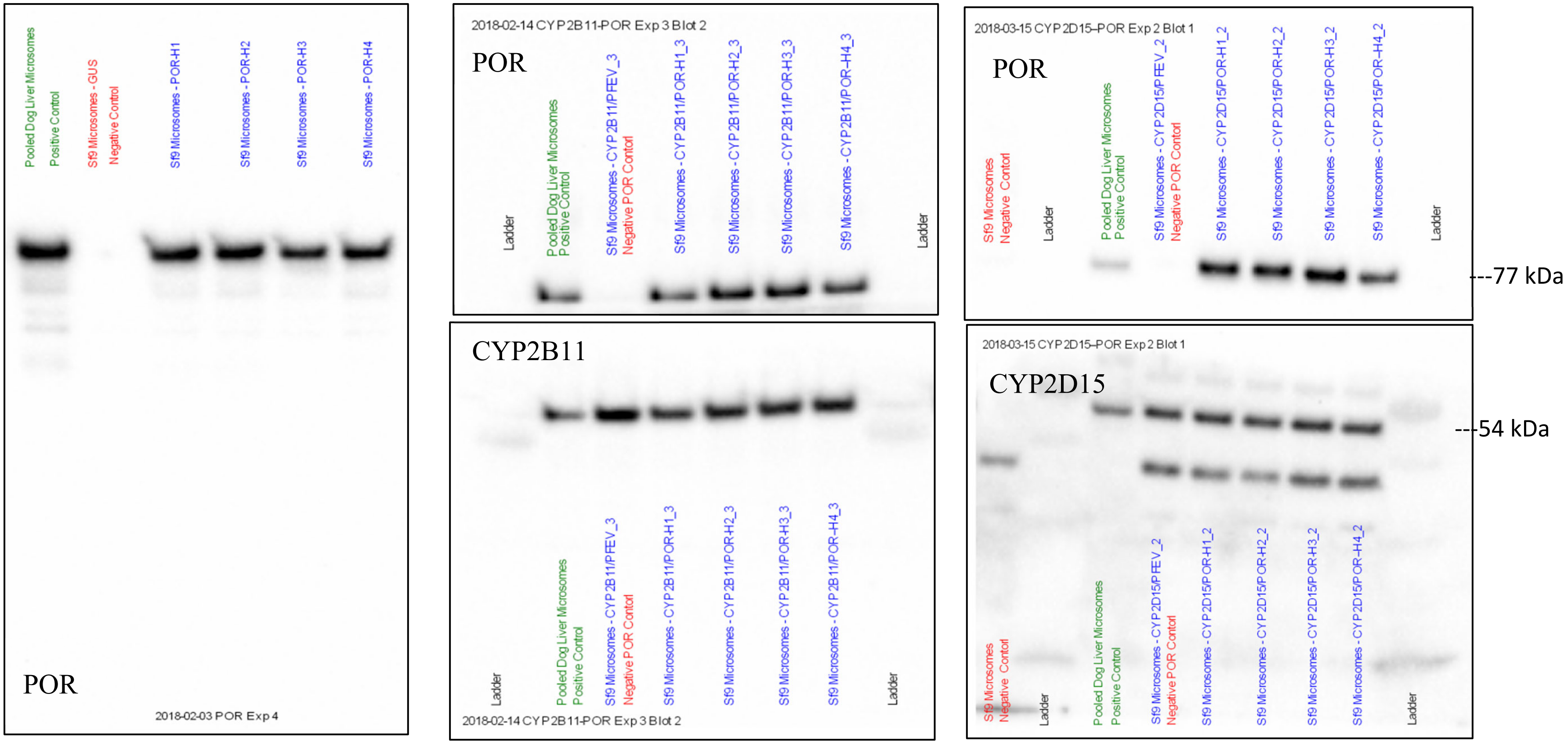

Supplement: S2 Fig — (PDF) [file pone.0297191.s002.pdf]
