# Supplementary material for: Pharmacogenomics of poor drug metabolism in greyhounds: Canine P450 oxidoreductase genetic variation, breed heterogeneity, and functional characterization
Source: PLoS One. 2024 Feb 1;19(2):e0297191. doi: 10.1371/journal.pone.0297191 (PMC10833530; doi:10.1371/journal.pone.0297191)

S3 Fig. Correlations between CYP2B11 catalytic activities using calculated V_{max} values (pmol/min/pmol CYP) and correlations between CYP2B11 catalytic activities (V_{max}) and cytochrome c reduction activities (nmol/min/mg microsomal protein) for recombinant microsomes. Also shown are the regression lines, the Spearman's correlation coefficients and P -values. (A) BROD and propofol as substrates; (B) BROD and bupropion as substrates; (C) propofol and bupropion as substrates; (D) cytochrome c and BROD as substrates; (E) cytochrome c and propofol as substrates; (F) cytochrome c and bupropion

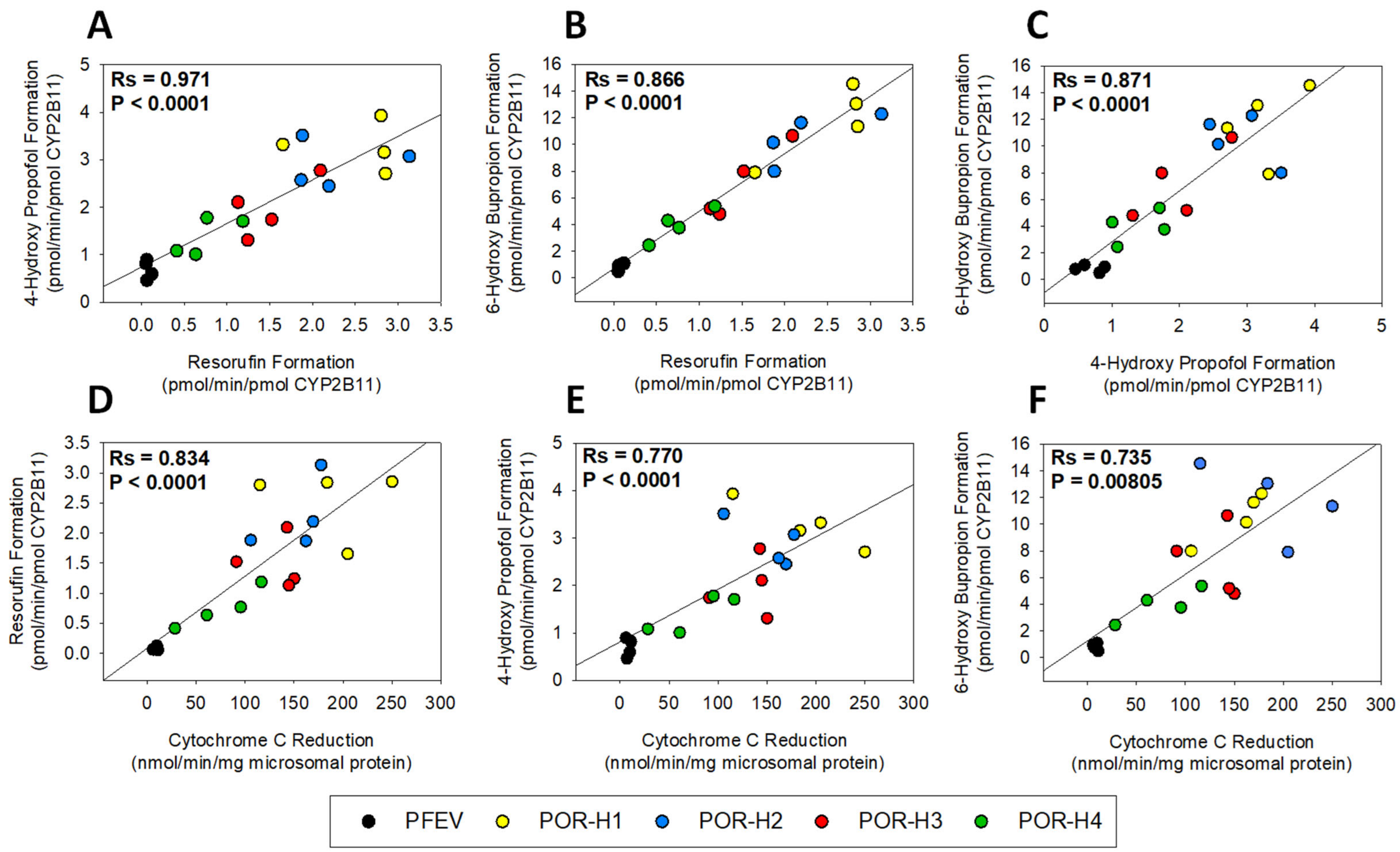

Supplement: S3 Fig — (PDF) [file pone.0297191.s003.pdf]
