# Supplementary material for: Pharmacogenomics of poor drug metabolism in greyhounds: Canine P450 oxidoreductase genetic variation, breed heterogeneity, and functional characterization
Source: PLoS One. 2024 Feb 1;19(2):e0297191. doi: 10.1371/journal.pone.0297191 (PMC10833530; doi:10.1371/journal.pone.0297191)

S4 Fig. Correlations between CYP2D15 catalytic activities using calculated V_{max} values (pmol/min/pmol P450) and correlations between CYP2D15 catalytic activities (V_{max}) and cytochrome c reduction activities (nmol/min/mg microsomal protein) for recombinant microsomes. Also shown are the regression lines, the Spearman's correlation coefficients and P - values. (A) tramadol and dextromethorphan as substrates; (B) cytochrome c and tramadol as substrates; (C) cytochrome c and dextromethorphan as substrates

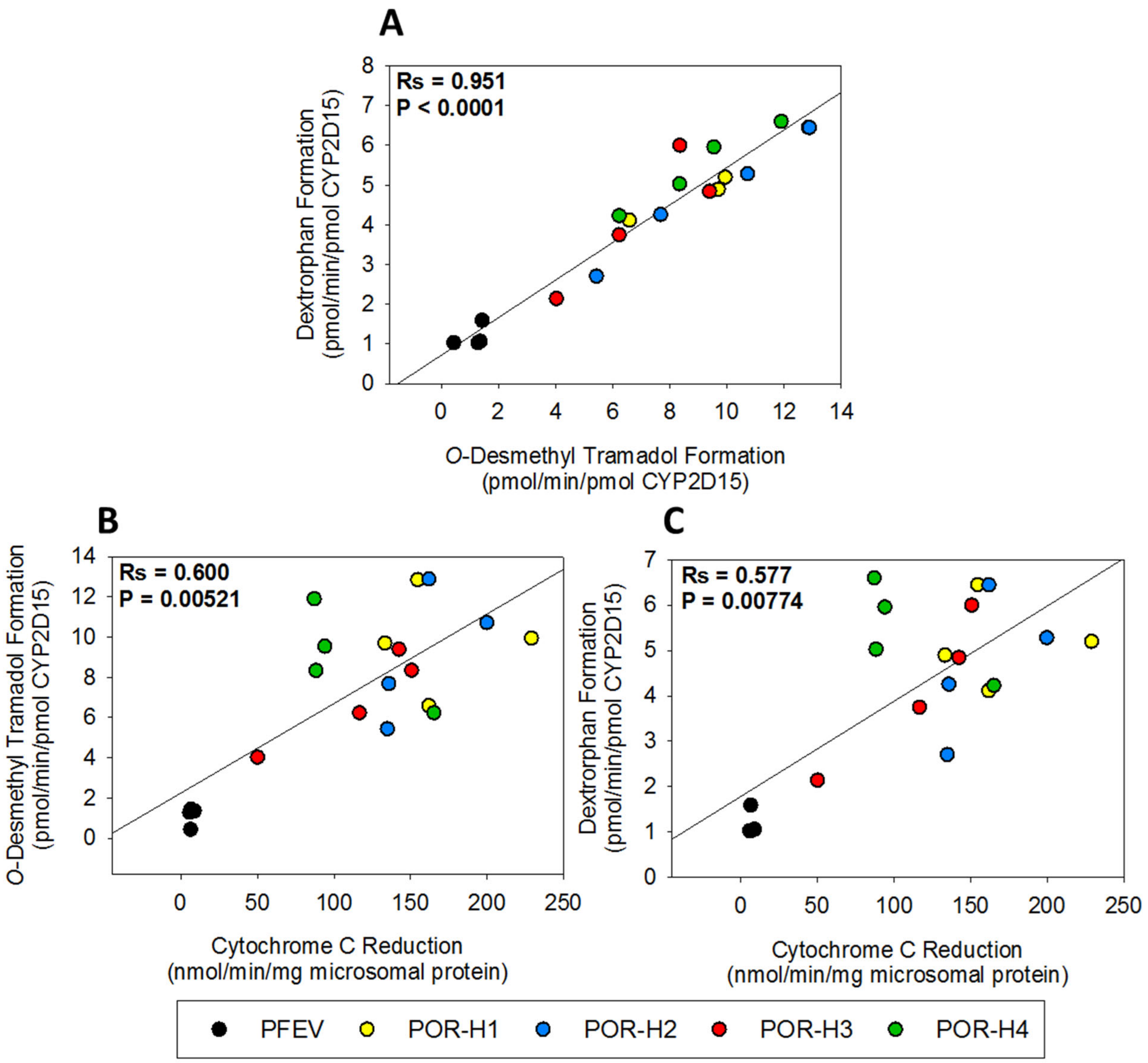

Supplement: S4 Fig — (PDF) [file pone.0297191.s004.pdf]
