# Supplementary material for: Pharmacogenomics of poor drug metabolism in greyhounds: Canine P450 oxidoreductase genetic variation, breed heterogeneity, and functional characterization
Source: PLoS One. 2024 Feb 1;19(2):e0297191. doi: 10.1371/journal.pone.0297191 (PMC10833530; doi:10.1371/journal.pone.0297191)
Supplement: S1 Table — (PDF) [file pone.0297191.s005.pdf]

S1 Table. *POR* gene PCR and sequencing primers. PCR primers used to amplify and sequence exons 1-16 of the *POR* gene from canine genomic DNA. *Indicates PCR primers that were also used to sequence the PCR product.

Primer ID	Sequence (5'-3')	Gene region amplified	Amplicon size (bp)
Pri_1196_forward*	CTTGCAGGCTTCAGGCTACT	Exon 1	207
Pri_1197_reverse	AGGCCCACAATGAGTGAAAA	Exon 1	207
Pri_1198_forward*	GTGGAGCAGCTCAGCCAGAG	Exon 2	202
Pri_1199_reverse	GTCCAGTGAAGGTGCCCTCC	Exon 2	202
Pri_1200_forward*	AGTCTCTGGTGAAGCCTGTG	Exon 3	190
Pri_1201_reverse	AGTCATATTCCTCGGGGTCT	Exon 3	190
Pri_1202_forward*	GTCTGTCTGCATCCCATCAG	Exon 4	217
Pri_1203_reverse	GAGAGGTCCACATCGGTCTC	Exon 4	217
Pri_1204_forward	GCACAAGATGTCTGACTACCAT	Exon 5	230
Pri_1205_reverse*	CACTCACTTTCCATCGTCATC	Exon 5	230
Pri_1206_forward*	CTTCAGTCACATCACTGGGC	Exon 6	322
Pri_1207_reverse	CAGTTTCTCAATGTGTCCCC	Exon 6	322
Pri_1208_forward*	CGTGTCCACTCGCTCTCCTTC	Exon 7	190
Pri_1209_reverse	CAACCACACTCTCCACCACTCAC	Exon 7	190
Pri_1210_forward*	GTGAAACCAGAAGGGTCCCT	Exons 8-9	429
Pri_1211_reverse	ACTCACCGTCAAGGTTGTTCA	Exons 8-9	429
Pri_1212_forward	TTGACGGTGAGTCCTGGAAT	Exons 10-11	556
Pri_1213_reverse*	GAGGCGATGGAGTAGTAGCG	Exons 10-11	556
Pri_1218_forward*	AGCAGGACCCCAGGATGGAG	Exon 12	227
Pri_1219_reverse	ACGAACATGGGCACCAGGGC	Exon 12	227
Pri_1220_forward*	TCTCTGGGGTCCCGTGAGGT	Exon 13	151
Pri_1221_reverse	TGGAACTGGGCCAGCTCCTC	Exon 13	151
Pri_1222_forward	CTCACAGGGTCTCTGCCTTC	Exon 14	244
Pri_1223_reverse*	AGAAGGTGTTCTGCACGTCC	Exon 14	244
Pri_1224_forward	TATGTCTGCGGGTGAGTGAG	Exon 15	310
Pri_1225_reverse*	AAAATGATTACACGGGGAGG	Exon 15	310
