# Supplementary material for: Pharmacogenomics of poor drug metabolism in greyhounds: Canine P450 oxidoreductase genetic variation, breed heterogeneity, and functional characterization
Source: PLoS One. 2024 Feb 1;19(2):e0297191. doi: 10.1371/journal.pone.0297191 (PMC10833530; doi:10.1371/journal.pone.0297191)
Supplement: S2 Table — (PDF) [file pone.0297191.s006.pdf]

S2 Table. Primer and reporter sequences used for Taqman allelic discrimination assay to genotype two *POR* single nucleotide polymorphisms.

Assay name	Polymorphism	Sequence (5'-3')	Purpose
POR-1	c.943 G/C	CCACCTGAGCCTCACATTCC	Forward primer
		GGTACACGGCCACATGGT	Reverse primer
		VIC-CCCAGGTAT**G**AATCTG-NFQ	Reporter 1
		FAM-CCCAGGTAT**C**AATCTG-NFQ	Reporter 2
POR-2	c.1710 C/G	CCGCCCAGGCAAGGA	Forward primer
		GCCGTCCTGGTGGAACTG	Reverse primer
		VIC-CAGGTAGTCCTC**G**TCAGAG-NFQ	Reporter 1
		FAM-CAGGTAGTCCTC**C**TCAGAG-NFQ	Reporter 2
