# Supplementary material for: Pharmacogenomics of poor drug metabolism in greyhounds: Canine P450 oxidoreductase genetic variation, breed heterogeneity, and functional characterization
Source: PLoS One. 2024 Feb 1;19(2):e0297191. doi: 10.1371/journal.pone.0297191 (PMC10833530; doi:10.1371/journal.pone.0297191)
Supplement: S3 Table — (PDF) [file pone.0297191.s007.pdf]

S3 Table. Genetic polymorphisms identified in the coding region of the POR gene in beagles and greyhounds. Polymorphisms were discovered and genotypes determined by PCR and Sanger sequencing of the entire coding region (exons 1 to 16) of the *POR* gene using DNA samples obtained from 5 beagles and 13 greyhounds. Samples from greyhounds were identified by their owners as dogs registered with the National Greyhound Association (NGA) bred for racing. Genotypes that differed from the reference sequence are highlighted.

	Position in Chromosome 6 (CanFam4):	7226481	7228851	7230963	7232297	7232326
	Gene location:	Exon 3	Exon 5	Exon 9	Exon 13	Exon 13
	cDNA change (NM_001177805):	c.244 T/C	c.576 G/A	c.943 G/C	c.1682 C/T	c.1710 C/G
	Amino acid change:	-	-	Glu315Gln	-	Asp570Glu
**Dog ID**	**Breed**					
DL15	Beagle	C/C	G/G	G/G	T/T	C/C
DL02	Beagle	T/C	G/G	G/G	C/T	C/C
DL20	Beagle	T/C	G/G	G/G	C/T	C/C
DL01	Beagle	C/C	G/G	G/G	T/T	C/C
DL16	Beagle	C/C	G/G	G/G	T/T	C/C
DL08	Greyhound	C/C	G/G	G/G	T/T	C/G
DL10	Greyhound	C/C	G/G	G/C	T/T	C/G
DL09	Greyhound	C/C	G/G	C/C	T/T	G/G
DL03	Greyhound	C/C	G/G	C/C	T/T	G/G
DL04	Greyhound	T/C	G/G	G/C	C/T	C/G
MC14-430	Greyhound	C/C	G/G	G/G	T/T	C/C
MC14-431	Greyhound	C/C	G/G	G/G	T/T	C/C
MC14-432	Greyhound	C/C	G/G	G/C	T/T	C/G
MC14-437	Greyhound	C/C	G/G	G/C	T/T	C/G
MC14-438	Greyhound	C/C	G/G	G/C	T/T	C/G
MC14-441	Greyhound	C/C	G/A	G/C	T/T	C/G
MC14-445	Greyhound	C/C	G/G	C/C	T/T	C/C
MC14-485	Greyhound	C/C	G/G	G/C	T/T	C/G
