# Supplementary material for: Pharmacogenomics of poor drug metabolism in greyhounds: Canine P450 oxidoreductase genetic variation, breed heterogeneity, and functional characterization
Source: PLoS One. 2024 Feb 1;19(2):e0297191. doi: 10.1371/journal.pone.0297191 (PMC10833530; doi:10.1371/journal.pone.0297191)
Supplement: S4 Table — (PDF) [file pone.0297191.s008.pdf]

S4 Table. Genotypes of the *POR* c.943 G/C and c.1710 C/G nonsynonymous SNPs determined by Taqman allelic discrimination assay using DNA sampled from 68 different dog breeds (21 sighhound and 47 non-sighthound breeds) and mixed-breed dogs. Shown are the numbers of dogs sampled from each breed, the numbers of dogs with each genotype and the minor allele frequencies for those polymorphisms.

		c.943 G/C genotype					c.1710 C/G genotype			
	N dogs	G/G	G/C	C/C	Allele %		C/C	C/G	G/G	Allele %
Sighthound breeds										
Afghan	31	29	2	0	3		31	0	0	0
Anatolian Shepherd	99	86	12	1	7		99	0	0	0
Azawakh	16	16	0	0	0		16	0	0	0
Basenji	27	24	1	0	2		27	0	0	0
Borzoi	57	39	14	1	15		57	0	0	0
Cirneco dell'Etna	19	15	4	0	11		15	4	0	11
Galgo Español	25	15	10	0	20		17	8	0	16
Greyhound-AKC**	64	50	14	0	11		50	14	0	11
Greyhound-NGA*	197	73	92	31	39		83	91	23	35
Ibizan Hound	19	19	0	0	0		19	0	0	0
Irish Wolfhound	27	27	0	0	0		27	0	0	0
Italian Greyhound	63	61	1	0	1		63	0	0	0
Windsprite	30	30	0	0	0		30	0	0	0
Peruvian Inca Orchid	21	21	0	0	0		21	0	0	0
Pharaoh Hound	26	25	0	0	0		26	0	0	0
Portuguese Podengo	12	10	2	0	8		11	1	0	4
Rhodesian Ridgeback	25	25	0	0	0		25	0	0	0
Saluki	14	9	4	1	21		13	1	0	4
Scottish Deerhound	168	73	70	25	36		74	67	27	36
Silken Windhound	51	51	0	0	0		51	0	0	0
Whippet	68	57	7	4	11		57	7	4	11
Non-sighthound breeds										
American Staffordshire Terrier	21	18	3	0	7		21	0	0	0
Australian Cattle Dog	13	13	0	0	0		13	0	0	0
Australian Shepherd	15	15	0	0	0		15	0	0	0
Basset Hound	10	10	0	0	0		10	0	0	0
Beagle	50	50	0	0	0		50	0	0	0
Bernese Mountain Dog	27	25	2	0	4		27	0	0	0
Border Collie	67	60	7	0	5		60	7	0	5
Boston Terrier	11	10	0	0	0		11	0	0	0
Boxer	20	20	0	0	0		20	0	0	0
Brittany Spaniel	26	26	0	0	0		26	0	0	0
Cairn Terrier	10	10	0	0	0		10	0	0	0

Cardigan Welsh Corgi	22	22	0	0	0		22	0	0	0
Chesapeake Bay Retriever	16	14	2	0	6		16	0	0	0
Chihuahua	14	13	1	0	4		14	0	0	0
Chow Chow	48	29	15	4	24		48	0	0	0
Cocker Spaniel	26	26	0	0	0		26	0	0	0
Collie	15	17	0	0	0		15	0	0	0
Dachshund	11	11	0	0	0		11	0	0	0
Doberman Pinscher	35	23	11	1	19		35	0	0	0
English Bulldog	20	20	0	0	0		20	0	0	0
French Bulldog	20	20	0	0	0		20	0	0	0
German Shepherd	42	42	0	0	0		42	0	0	0
German Shorthaired Pointer	13	13	0	0	0		13	0	0	0
Golden Retriever	62	62	0	0	0		62	0	0	0
Great Dane	10	10	0	0	0		10	0	0	0
Great Pyrenees	10	10	0	0	0		10	0	0	0
Jack Russell Terrier	12	11	1	0	4		12	0	0	0
Labrador Retriever	61	61	0	0	0		60	1	0	1
Miniature Dachshund	20	20	0	0	0		20	0	0	0
Miniature Poodle	10	10	0	0	0		10	0	0	0
Miniature Schnauzer	11	11	0	0	0		11	0	0	0
Newfoundland	10	10	0	0	0		10	0	0	0
Pembroke Welsh Corgi	22	22	0	0	0		22	0	0	0
Pitbull	34	32	2	0	3		34	0	0	0
Pomeranian	10	10	0	0	0		10	0	0	0
Pug	10	10	0	0	0		10	0	0	0
Rottweiler	42	9	22	10	51		42	0	0	0
Saint Bernard	23	21	2	0	4		23	0	0	0
Shetland Sheepdog	25	25	0	0	0		25	0	0	0
Shih Tzu	10	10	0	0	0		10	0	0	0
Siberian Husky	21	21	0	0	0		21	0	0	0
Soft-coated Wheaten Terrier	27	27	0	0	0		27	0	0	0
Springer Spaniel	11	11	0	0	0		11	0	0	0
Standard Poodle	21	21	0	0	0		21	0	0	0
Toy Poodle	20	20	0	0	0		20	0	0	0
Weimaraner	16	15	0	0	0		16	0	0	0
Yorkshire Terrier	29	29	0	0	0		29	0	0	0
Mixed breed dogs	168	149	16	3	7		163	5	0	1
