# Supplementary material for: Pharmacogenomics of poor drug metabolism in greyhounds: Canine P450 oxidoreductase genetic variation, breed heterogeneity, and functional characterization
Source: PLoS One. 2024 Feb 1;19(2):e0297191. doi: 10.1371/journal.pone.0297191 (PMC10833530; doi:10.1371/journal.pone.0297191)
Supplement: S1 File — (PDF) [file pone.0297191.s009.pdf]

<u>Raw data underlying all mean and standard deviation values presented in the manuscript</u>

S5 Fig. POR protein immunoblots of microsomes from Sf9 cells expressing wild-type POR (H1) or each POR variant (H2, H3 or H4).

S6 Fig. CYP2B11 and POR protein immunoblots of microsomes from Sf9 cells coexpressing CYP2B11 with wild-type POR (H1) or each POR variant (H2, H3 or H4).

S7 Fig. CYP2D15 and POR protein immunoblots of microsomes from Sf9 cells coexpressing CYP2D15 with wild-type POR (H1) or each POR variant (H2, H3 or H4).

S5 Table. Results of quantitation of POR protein immunoblots (shown in S3 Fig) of microsomes from Sf9 cells expressing wild-type POR (H1) or each POR variant (H2, H3 or H4).

S6 Table. Results of quantitation of POR and CYP2B11 protein immunoblots (shown in S4 Fig) of microsomes from Sf9 cells coexpressing CYP2B11 with wild-type POR (H1) or each POR variant (H2, H3 or H4).

S7 Table. Results of quantitation of POR and CYP2D15 protein immunoblots (shown in S5 Fig) of microsomes from Sf9 cells coexpressing CYP2D15 with wild-type POR (H1) or each POR variant (H2, H3 or H4).

S8 Table. Rates of microsomal cytochrome c reduction by POR-H1 and each POR variant (H2-H4) when cytochrome c concentration is varied and NADPH concentration is fixed.

S9 Table. Michaelis-Menten parameters Km, Vmax and CLint (intrinsic clearance; Vmax / Km) for microsomal cytochrome c reduction by POR-H1 and each POR variant (H2-H4) when cytochrome c concentration is varied and NADPH concentration is fixed.

S10 Table. Rates of microsomal cytochrome c reduction by POR-H1 and each POR variant (H2-H4) when NADPH concentration is varied and cytochrome c concentration is fixed.

S11 Table. Michaelis-Menten parameters Km, Vmax and CLint (intrinsic clearance; Vmax / Km) for microsomal cytochrome c reduction by POR-H1 and each POR variant (H2-H4) when NADPH concentration is varied and cytochrome c concentration is fixed.

S12 Table. Rates of microsomal cytochrome c reduction by POR-H1 and each POR variant (H2-H4) co-expressed with CYP2B11 or CYP2D15.

S13 Table. Rates of resorufin formation at different benzyloxyresorufin concentrations by CYP2B11 coexpressed with POR-H1, POR-H2, POR-H3, POR-H4, or pFastBac1 empty vector (PFEV) negative control in Sf9 microsomes.

S14 Table. Rates of 4-hydroxypropofol formation at different propofol concentrations by CYP2B11 coexpressed with POR-H1, POR-H2, POR-H3, POR-H4, or pFastBac1 empty vector (PFEV) negative control in Sf9 microsomes.

S15 Table. Rates of 6-hydroxybupropion formation at different bupropion concentrations by CYP2B11 coexpressed with POR-H1, POR-H2, POR-H3, POR-H4, or pFastBac1 empty vector (PFEV) negative control in Sf9 microsomes.

S16 Table. Rates of O-desmethyltramadol formation at different tramadol concentrations by CYP2D15 coexpressed with POR-H1, POR-H2, POR-H3, POR-H4, or pFastBac1 empty vector (PFEV) negative control in Sf9 microsomes.

S17 Table. Rates of dextrorphan formation at different dextromethorphan concentrations by CYP2D15 coexpressed with POR-H1, POR-H2, POR-H3, POR-H4, or pFastBac1 empty vector (PFEV) negative control in Sf9 microsomes.

S18 Table. Michaelis-Menten parameters Km, Vmax and CLint (intrinsic clearance; Vmax / Km) for microsomal resorufin formation at different benzyloxyresorufin concentrations by CYP2B11 coexpressed with POR-H1, POR-H2, POR-H3, POR-H4, or pFastBac1 empty vector (PFEV) negative control in Sf9 microsomes.

S19 Table. Michaelis-Menten parameters Km, Vmax and CLint (intrinsic clearance; Vmax / Km) for microsomal 4-hydroxypropofol formation at different propofol concentrations by CYP2B11 coexpressed with POR-H1, POR-H2, POR-H3, POR-H4, or pFastBac1 empty vector (PFEV) negative control in Sf9 microsomes.

S20 Table. Michaelis-Menten parameters Km, Vmax and CLint (intrinsic clearance; Vmax / Km) for microsomal 6-hydroxybupropion formation at different bupropion concentrations by CYP2B11 coexpressed with POR-H1, POR-H2, POR-H3, POR-H4, or pFastBac1 empty vector (PFEV) negative control in Sf9 microsomes.

S21 Table. Michaelis-Menten parameters Km, Vmax and CLint (intrinsic clearance; Vmax / Km) for microsomal O-desmethyltramadol formation at different tramadol concentrations by CYP2D15 coexpressed with POR-H1, POR-H2, POR-H3, POR-H4, or pFastBac1 empty vector (PFEV) negative control in Sf9 microsomes.

S5 Fig. POR protein immunoblots of microsomes from Sf9 cells expressing wild-type POR (H1) or each POR variant (H2, H3 or H4). Images of the same blots stained with Coomassie for total protein content are also shown. Tables on the left identify the samples loaded into each lane on the corresponding blot. Microsomal samples from each of four independently generated protein preparations (Experiment# 1-4) were blotted three times (Blot replicate # 1-3). β-glucuronidase (GUS) was used as the negative control and 1 or 3 ug of POR-H1 Sf9 microsomes (Std_1_ug; Std_3_ug) were used as the positive control. Other details are provided in the Materials and Methods section.

			Blot 1
	Experiment #	Blot replicate #	Lane
GUS_1			1
H1_1	1	1	2
H2_1	1	1	3
H3_1	1	1	4
H4_1	1	1	5
Std_1_ug			6
Std_3_ug			7
H1_2	2	1	8
H2_2	2	1	9
H3_2	2	1	10
H4_2	2	1	11
H1_3	3	1	12
H2_3	3	1	13
H3_3	3	1	14
H4_3	3	1	15

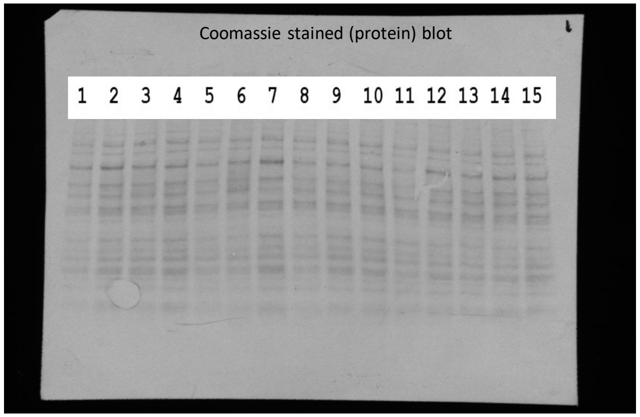

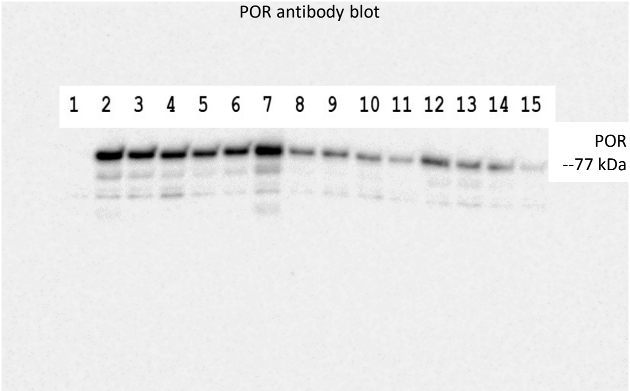

			Blot 2
	Experiment #	Blot replicate #	Lane
Std_1_ug			1
H1_4	4	1	2
H2_4	4	1	3
H3_4	4	1	4
H4_4	4	1	5
Std_3_ug			6
H1_1	1	2	7
H2_1	1	2	8
H3_1	1	2	9
H4_1	1	2	10
GUS_2			11
H1_2	2	2	12
H2_2	2	2	13
H3_2	2	2	14
H4_2	2	2	15

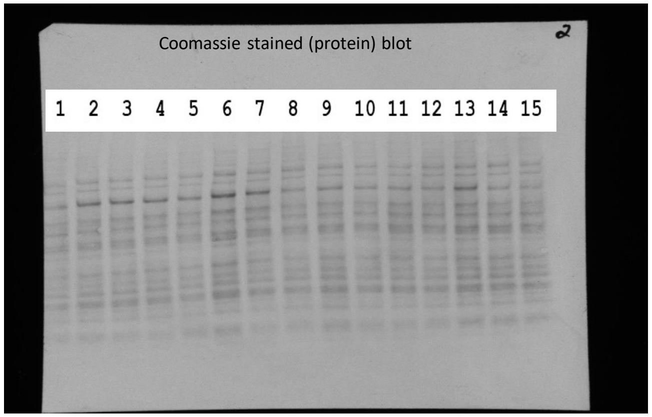

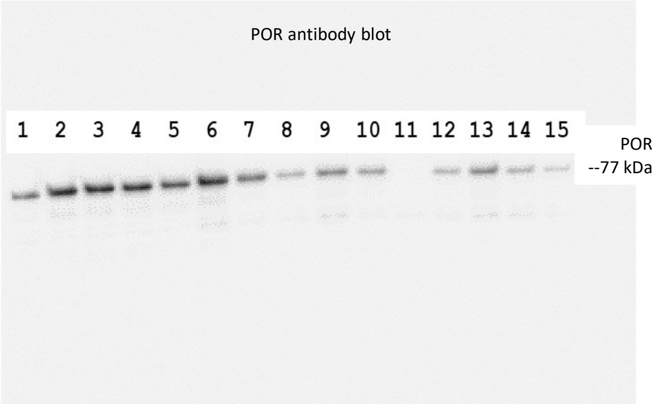

			Blot 3
	Experiment #	Blot replicate #	Lane
GUS_3			1
H1_3	3	2	2
H2_3	3	2	3
H3_3	3	2	4
H4_3	3	2	5
H1_4	4	2	6
H2_4	4	2	7
H3_4	4	2	8
H4_4	4	2	9
Std_1_ug			10
H1_1	1	3	11
H2_1	1	3	12
H3_1	1	3	13
H4_1	1	3	14
Std_3_ug			15

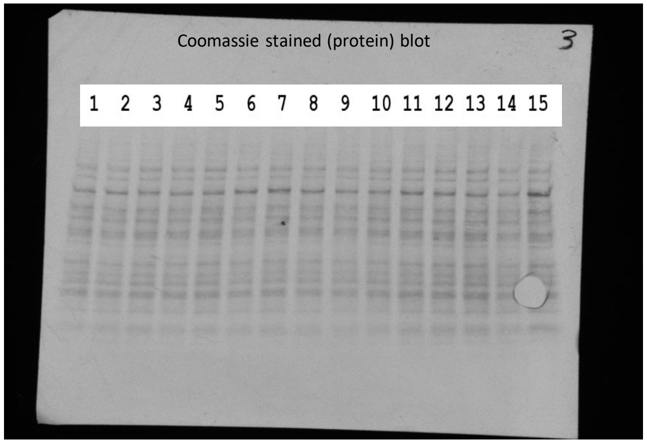

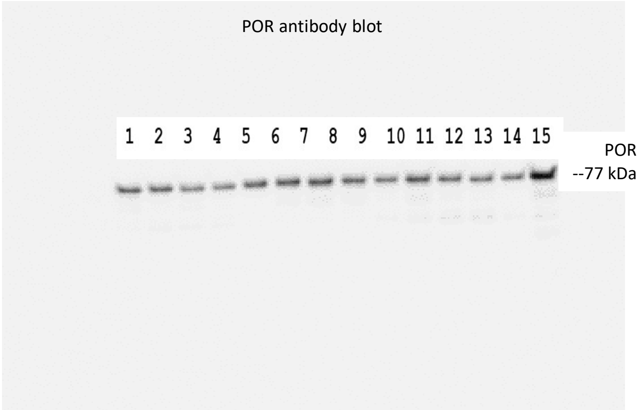

			Blot 4
	Experiment #	Blot replicate #	Lane
H1_2	2	3	1
H2_2	2	3	2
H3_2	2	3	3
H4_2	2	3	4
Std_1_ug			5
H1_3	3	3	6
H2_3	3	3	7
H3_3	3	3	8
H4_3	3	3	9
Std_3_ug			10
GUS_4			11
H1_4	4	3	12
H2_4	4	3	13
H3_4	4	3	14
H4_4	4	3	15

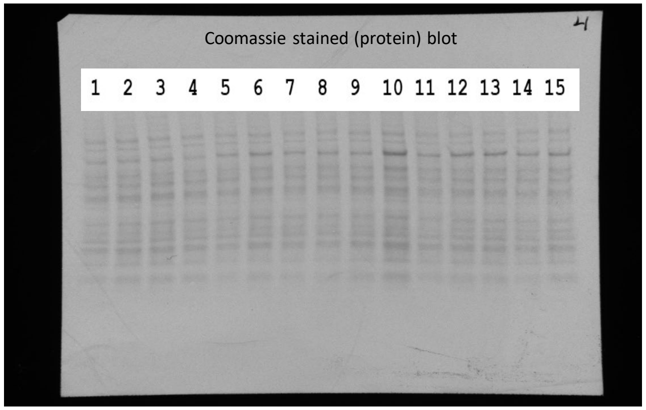

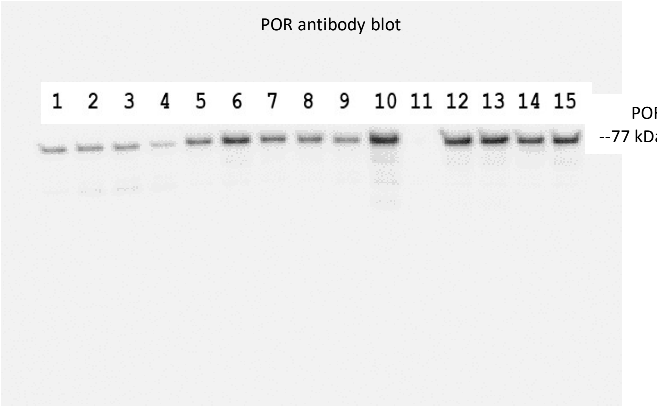

S6 Fig. CYP2B11 and POR protein immunoblots of microsomes from Sf9 cells coexpressing CYP2B11 with wild-type POR (H1) or each POR variant (H2, H3 or H4). Images of the same blots stained with Coomassie for total protein content are also shown. Tables to the left identify the samples loaded into each lane on the corresponding blot. Microsomal samples from each of four independently generated protein preparations (Experiment# 1-4) were blotted three times (Blot replicate # 1-3). Based on the significant size difference between the POR and CYP proteins, each blot was divided in half such that the top half contained POR (77 kDa) and the bottom half contained CYP2B11 (54 kDa). Each half was then incubated separately in the corresponding primary antibody, then together in the same secondary antibody, before chemiluminescence imaging. The reassembled top and botton halves are shown in the Coomassie stained blot image. pFastBac empty vector (PFEV) was used as the negative control and 1 or 3 ug of POR-H1 Sf9 microsomes (Std_1_ug; Std_3_ug) were used as positive controls. Other details are provided in the Materials and Methods section.

			Blot 1
	Experiment #	Blot replicate #	Lane
Ladder			1
PFEV_1	1	1	2
H1_1	1	1	3
H2_1	1	1	4
H3_1	1	1	5
H4_1	1	1	6
Std_1_ug			7
Ladder			8
Std_3_ug			9
PFEV_2	2	1	10
H1_2	2	1	11
H2_2	2	1	12
H3_2	2	1	13
H4_2	2	1	14
Ladder			15

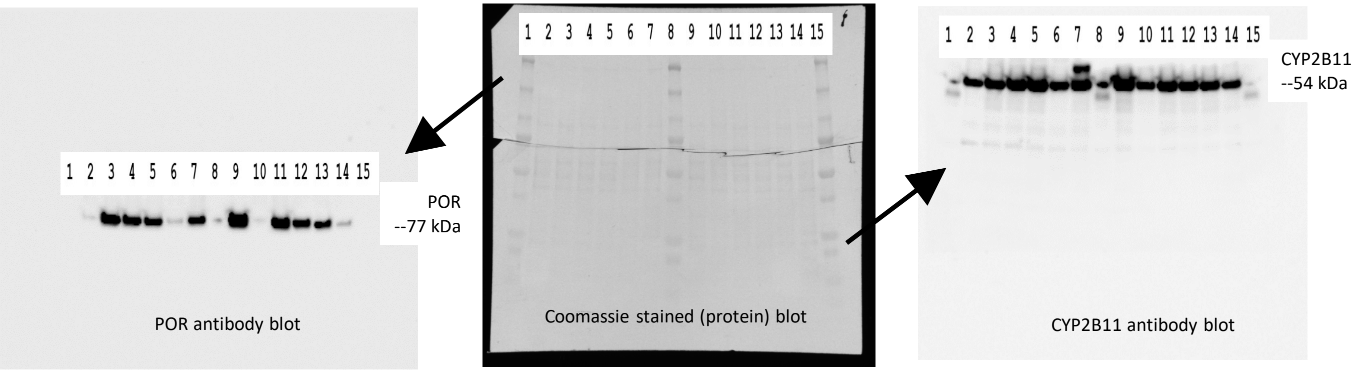

			Blot 2
	Experiment #	Blot replicate #	Lane
Ladder			1
Std_1_ug			2
PFEV_3	3	1	3
H1_3	3	1	4
H2_3	3	1	5
H3_3	3	1	6
H4_3	3	1	7
Ladder			8
PFEV_4	4	1	9
H1_4	4	1	10
H2_4	4	1	11
H3_4	4	1	12
H4_4	4	1	13
Std_3_ug			14
Ladder			15

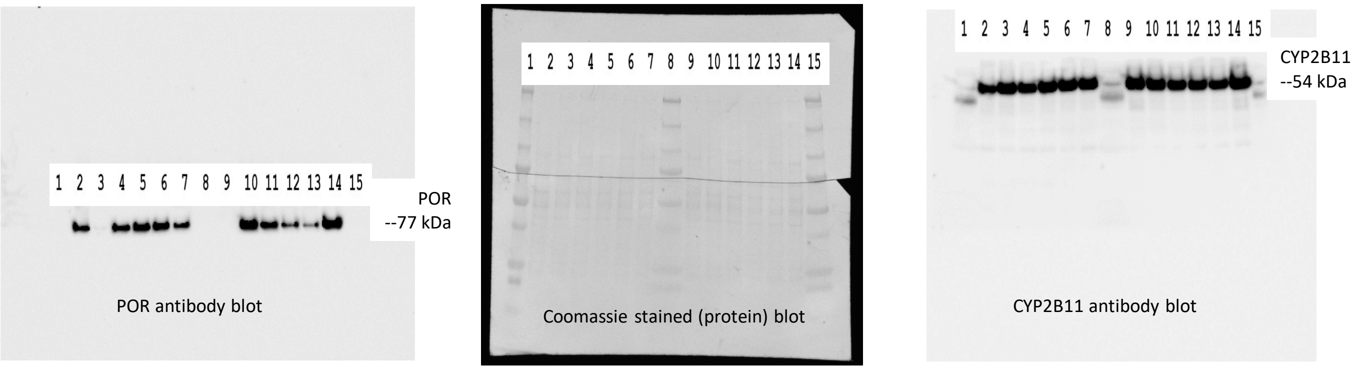

			Blot 3
	Experiment #	Blot replicate #	Lane
Ladder			1
Std_3_ug			2
Std_1_ug			3
PFEV_1	1	2	4
H1_1	1	2	5
H2_1	1	2	6
H3_1	1	2	7
H4_1	1	2	8
Ladder			9
PFEV_2	2	2	10
H1_2	2	2	11
H2_2	2	2	12
H3_2	2	2	13
H4_2	2	2	14
Ladder			15

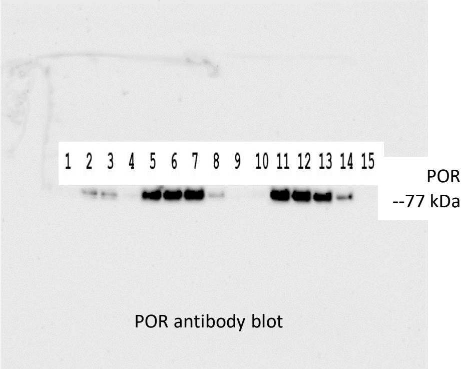

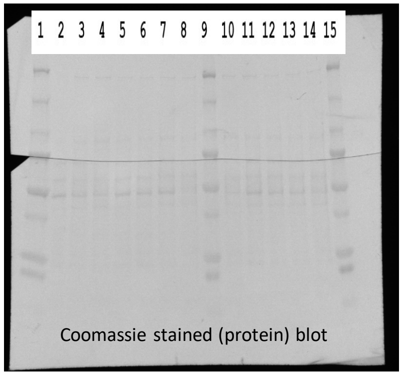

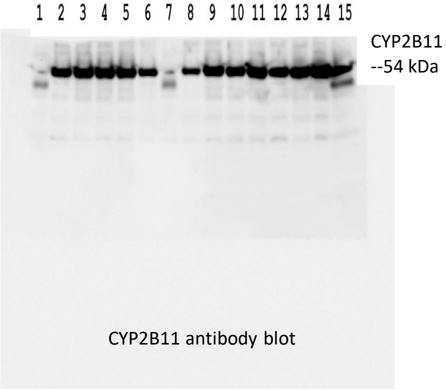

			Blot 4
	Experiment #	Blot replicate #	Lane
Ladder			1
PFEV_3	3	2	2
H1_3	3	2	3
H2_3	3	2	4
H3_3	3	2	5
H4_3	3	2	6
Ladder			7
PFEV_4	4	2	8
H1_4	4	2	9
H2_4	4	2	10
H3_4	4	2	11
H4_4	4	2	12
Std_1_ug			13
Std_3_ug			14
Ladder			15

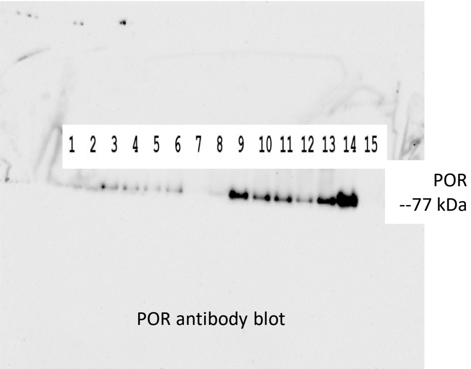

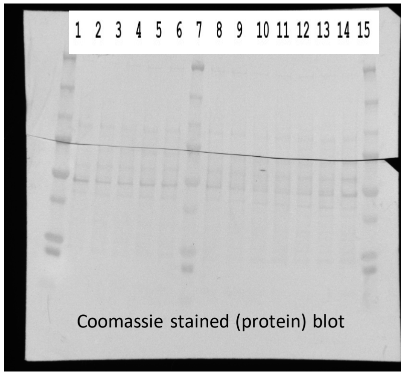

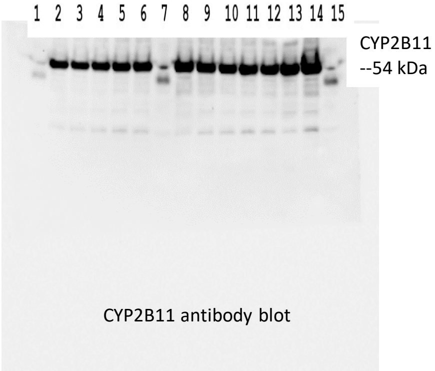

			Blot 5
	Experiment #	Blot replicate #	Lane
Ladder			1
PFEV_1	1	3	2
H1_1	1	3	3
H2_1	1	3	4
H3_1	1	3	5
H4_1	1	3	6
Ladder			7
PFEV_2	2	3	8
H1_2	2	3	9
H2_2	2	3	10
H3_2	2	3	11
H4_2	2	3	12
Std_3_ug			13
Std_1_ug			14
Ladder			15

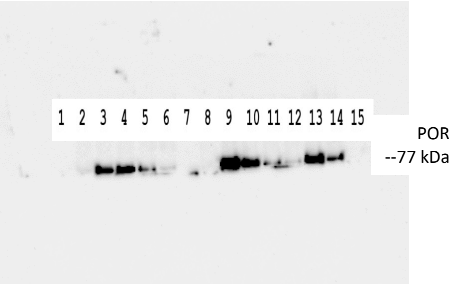

POR antibody blot

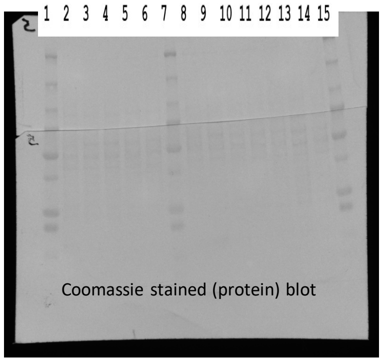

Coomassie stained (protein) blot

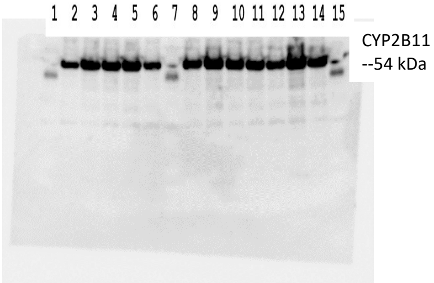

CYP2B11 antibody blot

			Blot 6
	Experiment #	Blot replicate #	Lane
Ladder			1
Std_3_ug			2
PFEV_3	3	3	3
H1_3	3	3	4
H2_3	3	3	5
H3_3	3	3	6
H4_3	3	3	7
Ladder			8
Std_1ug			9
PFEV_4	4	3	10
H1_4	4	3	11
H2_4	4	3	12
H3_4	4	3	13
H4_4	4	3	14
Ladder			15

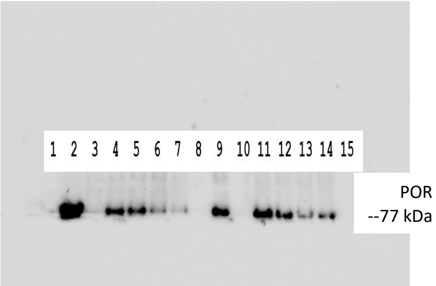

POR antibody blot

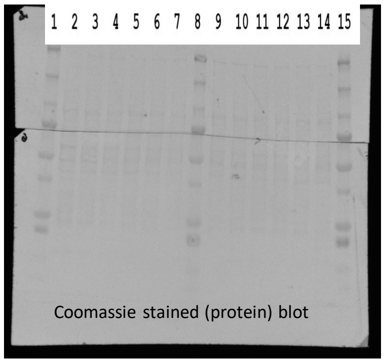

Coomassie stained (protein) blot

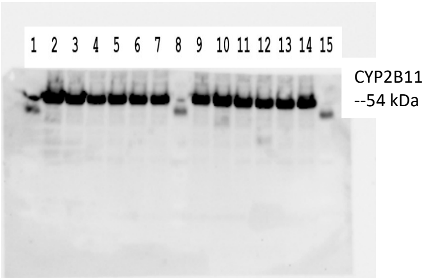

CYP2B11 antibody blot

S7 Fig. CYP2D15 and POR protein immunoblots of microsomes from Sf9 cells coexpressing CYP2D15 with wild-type POR (H1) or each POR variant (H2, H3 or H4). Tables to the left identify the samples loaded into each lane on the corresponding blot. Microsomal samples from each of four independently generated protein preparations (Experiment# 1-4) were blotted three times (Blot replicate # 1-3). Based on the significant size difference between the POR and CYP proteins, each blot was divided in half such that the top half contained POR (77 kDa) and the bottom half contained CYP2D15 (54 kDa). Each half was then incubated separately in the corresponding primary antibody, then together in the same secondary antibody, before chemiluminescence imaging. The top and botton halves are shown in the Coomassie stained blot image. pFastBac empty vector (PFEV) was used as a POR negative control and microsomes from uninfected Sf9 cells were used as a POR and CYP2D15 negative control. Pooled dog liver microsomes (pDLMs) were used as a POR and CYP2D15 positive control. Other details are provided in the Materials and Methods section. In the CYP2D15 blots, note a prominent band that is faster migrating (about 10 kDa smaller) than the 54 kDa CYP2D15 band. This band is also found in uninfected Sf9 cells, but absent from pDLMs suggesting that it is a crossreactive background protein expressed in Sf9 cells.

			Blot 1
	Experiment #	Blot replicate #	Lane
Ladder			1
PFEV_1	1	1	2
H1_1	1	1	3
H2_1	1	1	4
H3_1	1	1	5
H4_1	1	1	6
Sf9 cells			7
Ladder			8
pDLMs			9
PFEV_2	2	1	10
H1_2	2	1	11
H2_2	2	1	12
H3_2	2	1	13
H4_2	2	1	14
Ladder			15

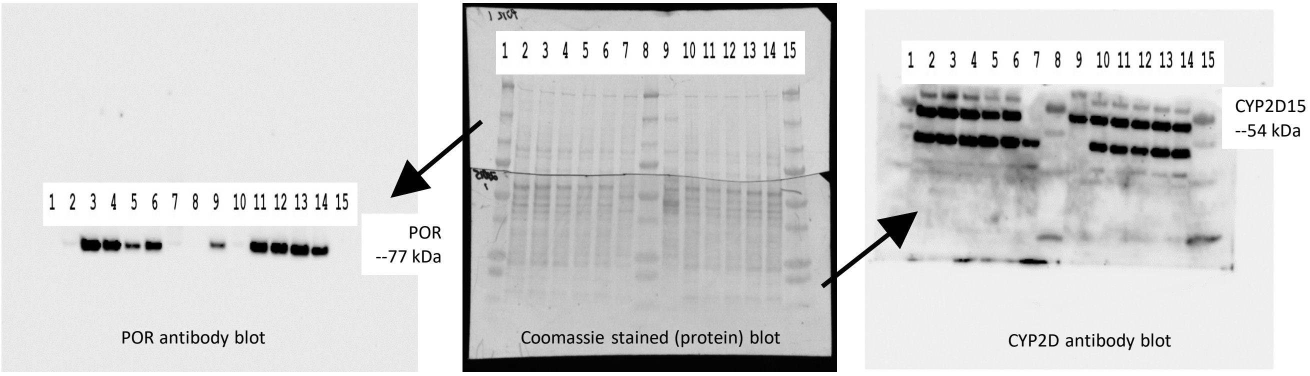

			Blot 2
	Experiment #	Blot replicate #	Lane
Ladder			1
Sf9 cells			2
PFEV_3	3	1	3
H1_3	3	1	4
H2_3	3	1	5
H3_3	3	1	6
H4_3	3	1	7
Ladder			8
PFEV_4	4	1	9
H1_4	4	1	10
H2_4	4	1	11
H3_4	4	1	12
H4_4	4	1	13
pDLMs			14
Ladder			15

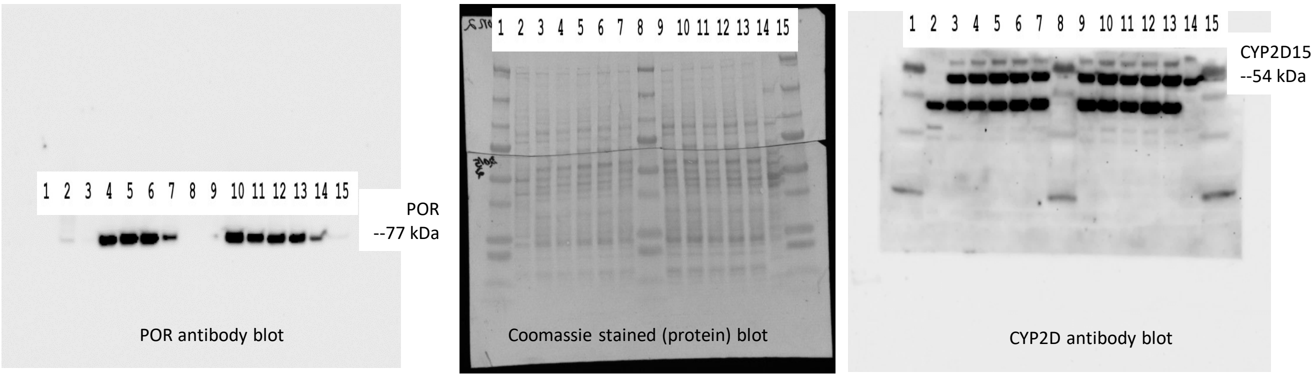

			Blot 3
	Experiment #	Blot replicate #	Lane
Ladder			1
pDLMs			2
PFEV_1	1	2	3
H1_1	1	2	4
H2_1	1	2	5
H3_1	1	2	6
H4_1	1	2	7
Ladder			8
Sf9 cells			9
PFEV_2	2	2	10
H1_2	2	2	11
H2_2	2	2	12
H3_2	2	2	13
H4_2	2	2	14
Ladder			15

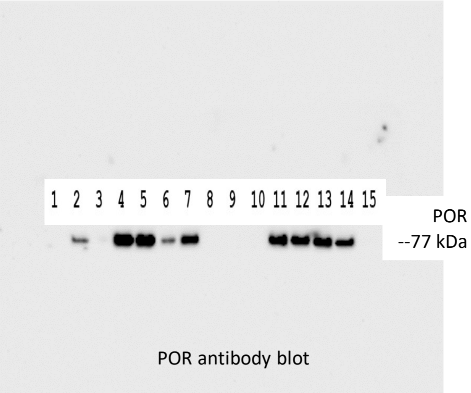

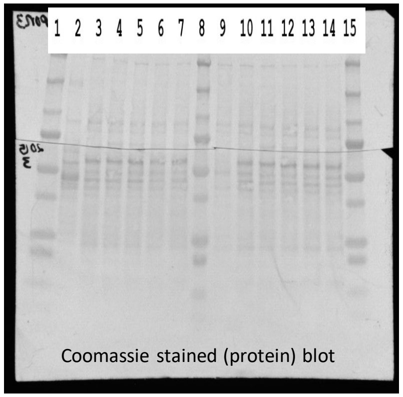

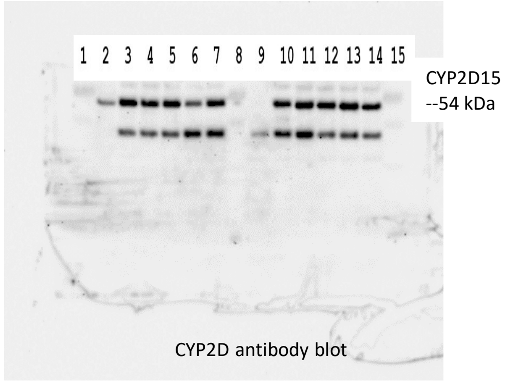

			Blot 4
	Experiment #	Blot replicate #	Lane
Ladder			1
PFEV_3	3	2	2
H1_3	3	2	3
H2_3	3	2	4
H3_3	3	2	5
H4_3	3	2	6
Sf9 cells			7
Ladder			8
PFEV_4	4	2	9
H1_4	4	2	10
H2_4	4	2	11
H3_4	4	2	12
H4_4	4	2	13
pDLMs		2	14
Ladder		2	15

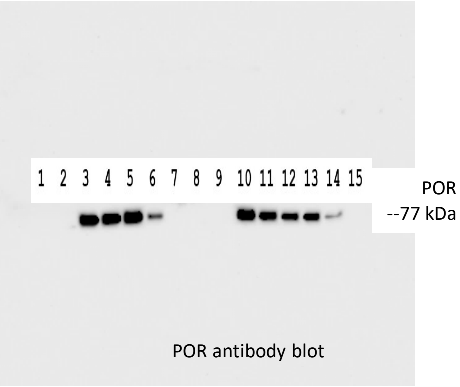

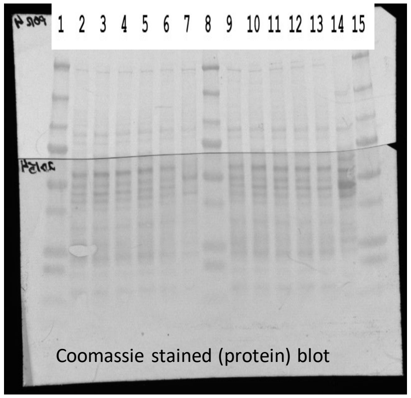

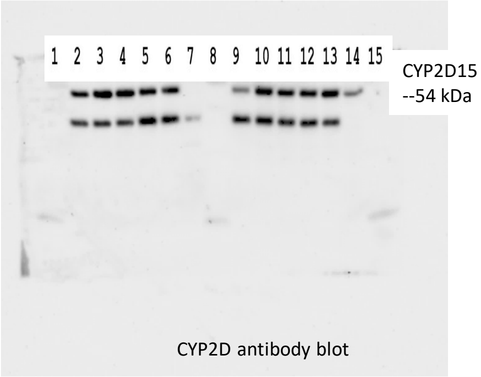

			Blot 5
	Experiment #	Blot replicate #	Lane
Ladder			1
PFEV_1	1	3	2
H1_1	1	3	3
H2_1	1	3	4
H3_1	1	3	5
H4_1	1	3	6
pDLMs			7
Ladder			8
PFEV_2	2	3	9
H1_2	2	3	10
H2_2	2	3	11
H3_2	2	3	12
H4_2	2	3	13
Sf9 cells			14
Ladder			15

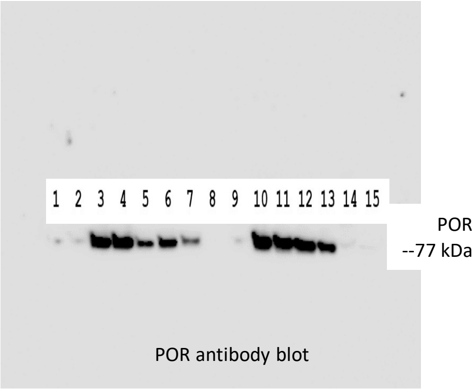

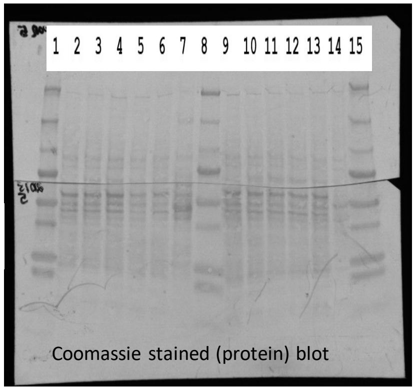

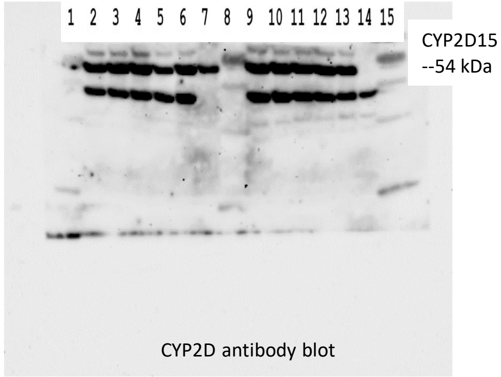

			Blot 6
	Experiment #	Blot replicate #	Lane
Ladder			1
Sf9 cells			2
PFEV_3	3	3	3
H1_3	3	3	4
H2_3	3	3	5
H3_3	3	3	6
H4_3	3	3	7
Ladder			8
pDLMs			9
PFEV_4	4	3	10
H1_4	4	3	11
H2_4	4	3	12
H3_4	4	3	13
H4_4	4	3	14
Ladder			15

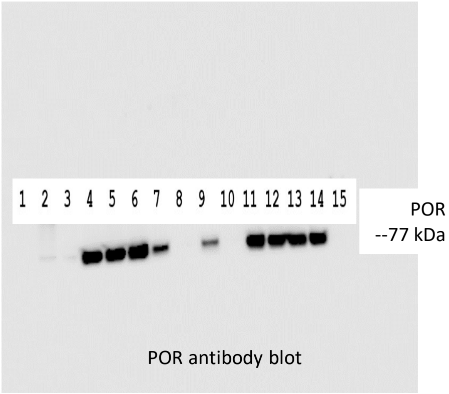

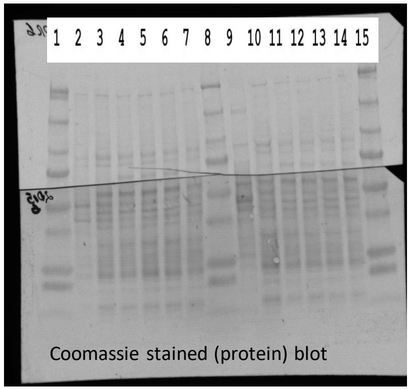

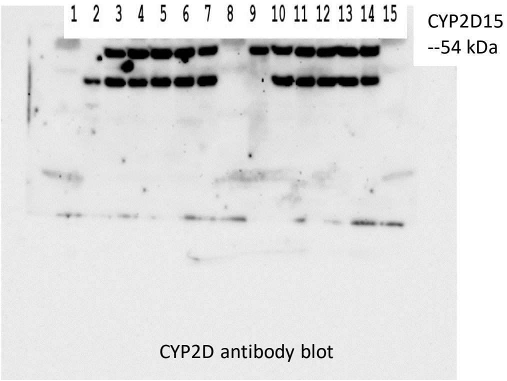

S5 Table. Results of quantitation of POR protein immunoblots (shown in S3 Fig) of microsomes from Sf9 cells expressing wild-type POR (H1) or each POR variant (H2, H3 or H4). Microsomal samples from each of four independently generated protein preparations (Experiment # 1-4) were blotted three times. The results below are the average of the total protein normalized band intensity for the 3 replicate blots in each protein preparation. Other details are provided in the Materials and Methods section. SD – standard deviation CV% - coefficient of variation as a percentage.

	POR protein band pixel volume normalized to total protein stained lane pixel volume (ratio)						
	Experiment #						
POR variant	Exp1	Exp2	Exp3	Exp4	Mean	SD	CV%
H1	218	222	213	249	225	16	7
H2	203	197	212	245	214	21	10
H3	211	195	211	239	214	18	9
H4	229	214	194	255	223	26	12

S6 Table. Results of quantitation of POR and CYP2B11 protein immunoblots (shown in S4 Fig) of microsomes from Sf9 cells coexpressing CYP2B11 with wild-type POR (H1) or each POR variant (H2, H3 or H4). Microsomal samples from each of four independently generated protein preparations (Experiment # 1-4) were blotted three times. The results below are the average of the total protein normalized band intensity for the 3 replicate blots in each protein preparation. Other details are provided in the Materials and Methods section. SD – standard deviation CV% - coefficient of variation.

	POR protein band pixel volume normalized to total protein stained lane pixel volume (ratio)						
	Experiment #						
POR variant	Exp1	Exp2	Exp3	Exp4	Mean	SD	CV%
PFEV (vector control)	0	0	0	0	0	0	0
H1	386	394	337	292	352	48	14
H2	362	416	303	280	340	61	18
H3	382	393	277	271	331	66	20
H4	385	342	282	234	311	66	21
	CYP2B11 protein band pixel volume normalized to total protein stained lane pixel volume (ratio)						
	Experiment #						
POR variant	Exp1	Exp2	Exp3	Exp4	Mean	SD	CV%
PFEV (vector control)	442	454	369	424	422	38	9
H1	481	395	345	345	392	64	16
H2	416	463	366	387	408	42	10
H3	488	461	353	365	417	68	16
H4	435	435	377	330	394	51	13

S7 Table. Results of quantitation of POR and CYP2D15 protein immunoblots (shown in S5 Fig) of microsomes from Sf9 cells coexpressing CYP2D15 with wild-type POR (H1) or each POR variant (H2, H3 or H4). Microsomal samples from each of four independently generated protein preparations (Experiment # 1-4) were blotted three times. The results below are the average of the total protein normalized band intensity for the 3 replicate blots in each protein preparation. Other details are provided in the Materials and Methods section. SD – standard deviation CV% - coefficient of variation.

	POR protein band volume normalized to total protein stained lane volume (ratio)						
	Experiment #						
POR variant	Exp1	Exp2	Exp3	Exp4	Mean	SD	CV%
PFEV (vector control)	0	0	0	0	0	0	0
H1	218	242	241	199	225	21	9
H2	215	236	234	208	223	14	6
H3	214	155	212	221	200	31	15
H4	216	151	232	179	195	37	19
	CYP2B11 protein band volume normalized to total protein stained lane volume (ratio)						
	Experiment #						
POR variant	Exp1	Exp2	Exp3	Exp4	Mean	SD	CV%
PFEV (vector control)	196	217	169	153	184	29	16
H1	214	242	219	190	217	21	10
H2	216	226	222	200	216	11	5
H3	233	219	178	222	213	24	11
H4	237	158	222	182	200	36	18

S8 Table. Results of an enzyme kinetic study evaluating the effect of POR mutations on cytochrome c reduction by POR expressed in Sf9 cells. Shown are rates of microsomal cytochrome c reduction by POR-H1 and each POR variant (H2-H4) when cytochrome c concentration is varied and NADPH concentration is fixed. Microsomal samples from each of four independently generated protein preparations (Experiment # 1-4) were independently tested three times each. The activities shown below are the average of 3 independent incubations of each protein preparation. Other details are provided in the Materials and Methods section. SD – standard deviation

POR-H1	nmole Cytochrome C reduced/min/mg protein					
uM Cytochrome C	Exp1	Exp2	Exp3	Exp4	Mean	SD
0	0.0	0.0	0.0	0.0	0.0	0.0
2.5	90.6	65.6	69.3	76.7	75.6	11.0
5	178.1	112.2	136.1	139.8	141.5	27.2
10	314.8	185.6	226.5	238.4	241.3	54.0
15	408.1	233.1	284.8	297.7	305.9	73.6
30	588.4	308.9	392.0	400.2	422.4	118.1
50	711.5	374.8	451.1	465.6	500.8	146.0
75	725.8	379.0	454.8	511.9	517.9	148.9
100	783.8	411.4	507.4	523.5	556.5	159.4
POR-H2	nmole Cytochrome C reduced/min/mg protein					
uM Cytochrome C	Exp1	Exp2	Exp3	Exp4	Mean	SD
0	0.0	0.0	0.0	0.0	0.0	0.0
2.5	82.0	45.0	58.0	93.1	69.5	21.9
5	149.2	76.8	112.1	161.4	124.9	38.3
10	246.8	120.2	188.6	279.6	208.8	70.0
15	329.7	148.7	222.5	358.8	264.9	97.1
30	438.0	199.2	304.4	476.0	354.4	127.0
50	517.3	210.7	364.4	551.8	411.1	156.5
75	566.3	248.4	372.0	584.7	442.8	161.4
100	568.5	255.1	376.5	626.8	456.7	171.8
POR-H3	nmole Cytochrome C reduced/min/mg protein					
uM Cytochrome C	Exp1	Exp2	Exp3	Exp4	Mean	SD
0	0.0	0.0	0.0	0.0	0.0	0.0
2.5	55.1	44.1	61.7	72.4	58.3	11.8
5	104.8	78.8	115.3	135.9	108.7	23.7
10	172.7	132.1	201.8	210.9	179.4	35.4
15	211.8	159.6	243.4	278.6	223.4	50.5
30	296.0	221.2	329.3	373.9	305.1	64.4
50	320.8	253.9	373.5	434.9	345.8	77.0
75	350.8	269.7	399.6	462.1	370.5	81.2
100	357.3	295.5	412.0	507.5	393.1	89.9

POR-H4	nmole Cytochrome C reduced/min/mg protein					
uM Cytochrome C	Exp1	Exp2	Exp3	Exp4	Mean	SD
0	0.0	0.0	0.0	0.0	0.0	0.0
2.5	56.7	37.4	47.7	74.9	54.2	15.9
5	117.4	61.4	88.7	132.9	100.1	31.6
10	247.6	102.8	151.9	229.5	183.0	67.6
15	310.5	129.1	192.9	299.4	233.0	87.2
30	389.0	173.5	266.7	414.6	311.0	112.1
50	470.1	203.6	293.6	474.9	360.6	134.4
75	519.9	217.5	313.0	529.1	394.9	154.7
100	519.9	237.0	315.9	546.2	404.8	152.0

S9 Table. Results of an enzyme kinetic study evaluating the effect of POR mutations on cytochrome c reduction by POR expressed in Sf9 cells. Shown are Michaelis-Menten parameters Km, Vmax and CLint (intrinsic clearance; Vmax / Km) for microsomal cytochrome c reduction by POR-H1 and each POR variant (H2-H4) when cytochrome c concentration is varied and NADPH concentration is fixed. Enzyme kinetic parameters were derived by fitting a one-enzyme Michaelis-Menten model to measured enzyme activities and substrate concentrations (data in Table S8) using nonlinear regression. Microsomal samples from each of four independently generated protein preparations (Experiment # 1-4) were independently tested three times each. The parameters shown below are the average of 3 independent incubations of each protein preparation. Other details are provided in the Materials and Methods section. SD – standard deviation

	POR-H1					
	Exp1	Exp2	Exp3	Exp4	Mean	SD
Km (uM)	19.7	15.5	15.5	16.2	16.7	2.0
Vmax (nmol/min/mg protein)	947.1	472.5	577.4	616.1	653.3	205.1
CLint (mL/min/mg protein)	48.1	30.5	37.2	37.9	38.5	7.3
	POR-H2					
	Exp1	Exp2	Exp3	Exp4	Mean	SD
Km (uM)	16.9	14.0	14.2	14.2	14.8	1.4
Vmax (nmol/min/mg protein)	682.9	287.4	444.6	684.9	524.9	194.4
CLint (mL/min/mg protein)	40.3	20.5	31.3	48.2	35.1	11.9
	POR-H3					
	Exp1	Exp2	Exp3	Exp4	Mean	SD
Km (uM)	14.1	16.1	14.4	16.9	15.4	1.3
Vmax (nmol/min/mg protein)	415.0	336.4	477.7	581.8	452.7	103.7
CLint (mL/min/mg protein)	29.4	20.9	33.1	34.4	29.5	6.1
	POR-H4					
	Exp1	Exp2	Exp3	Exp4	Mean	SD
Km (uM)	17.1	16.6	14.3	18.2	16.5	1.6
Vmax (nmol/min/mg protein)	624.9	271.4	372.8	652.3	480.3	187.7
CLint (mL/min/mg protein)	36.6	16.3	26.1	35.9	28.7	9.6

S10 Table. Results of an enzyme kinetic study evaluating the effect of POR mutations on cytochrome c reduction by POR expressed in Sf9 cells. Shown are rates of microsomal cytochrome c reduction by POR-H1 and each POR variant (H2-H4) when NADPH concentration is varied and cytochrome c concentration is fixed. Microsomal samples from each of four independently generated protein preparations (Experiment # 1-4) were independently tested three times each. The activities shown below are the average of 3 independent incubations of each protein preparation. Other details are provided in the Materials and Methods section. SD – standard deviation

POR-H1	nmole Cytochrome C reduced/min/mg protein					
uM NADPH	Exp1	Exp2	Exp3	Exp4	Mean	SD
0	0.0	0.0	0.0	0.0	0.0	0.0
1.5625	149.5	77.5	116.1	141.8	121.2	32.4
3.125	255.1	104.6	152.4	191.8	176.0	63.7
6.25	310.8	134.6	198.9	261.1	226.4	76.4
12.5	396.1	156.5	250.5	310.3	278.3	100.9
25	473.8	178.1	277.1	359.2	322.0	125.4
50	478.7	209.0	296.1	388.2	343.0	116.4
75	470.3	195.2	304.4	355.6	331.4	114.2
POR-H2	nmole Cytochrome C reduced/min/mg protein					
uM NADPH	Exp1	Exp2	Exp3	Exp4	Mean	SD
0	0.0	0.0	0.0	0.0	0.0	0.0
1.5625	117.7	66.4	117.0	164.6	116.4	40.1
3.125	186.1	91.5	167.9	277.6	180.8	76.5
6.25	257.2	124.1	195.8	404.3	245.4	119.1
12.5	278.3	136.3	235.6	464.5	278.7	137.4
25	337.6	151.6	270.8	534.2	323.5	160.1
50	360.1	176.9	316.0	590.2	360.8	171.7
75	369.1	164.4	286.2	569.0	347.2	170.1
POR-H3	nmole Cytochrome C reduced/min/mg protein					
uM NADPH	Exp1	Exp2	Exp3	Exp4	Mean	SD
0	0.0	0.0	0.0	0.0	0.0	0.0
1.5625	75.2	64.4	84.8	121.1	86.4	24.6
3.125	112.8	92.0	145.7	163.3	128.5	32.1
6.25	150.0	118.3	178.9	245.6	173.2	54.3
12.5	162.8	142.5	199.9	291.6	199.2	66.0
25	171.8	155.1	225.6	290.1	210.6	60.9
50	188.4	178.8	230.2	332.7	232.5	70.4
75	193.2	172.2	248.4	328.0	235.4	69.6

POR-H4	nmole Cytochrome C reduced/min/mg protein					
uM NADPH	Exp1	Exp2	Exp3	Exp4	Mean	SD
0	0.0	0.0	0.0	0.0	0.0	0.0
1.5625	88.8	55.8	134.0	119.9	99.6	34.8
3.125	131.7	77.8	180.6	160.9	137.7	44.7
6.25	168.8	93.8	181.1	225.8	167.4	54.8
12.5	208.2	106.1	236.9	250.3	200.4	65.2
25	222.7	122.9	265.7	271.2	220.6	68.6
50	240.6	130.0	292.6	305.3	242.1	79.8
75	231.9	131.9	279.1	292.7	233.9	72.8

S11 Table. Results of an enzyme kinetic study evaluating the effect of POR mutations on cytochrome c reduction by POR expressed in Sf9 cells. Shown are Michaelis-Menten parameters Km, Vmax and CLint (intrinsic clearance; Vmax / Km) for microsomal cytochrome c reduction by POR-H1 and each POR variant (H2-H4) when NADPH concentration is varied and cytochrome c concentration is fixed. Enzyme kinetic parameters were derived by fitting a one-enzyme Michaelis-Menten model to measured enzyme activities and substrate concentrations (data in Table S10) using nonlinear regression. Microsomal samples from each of four independently generated protein preparations (Experiment # 1-4) were independently tested three times each. The parameters shown below are the average of 3 independent incubations of each protein preparation. Other details are provided in the Materials and Methods section. SD – standard deviation.

	POR-H1					
	Exp1	Exp2	Exp3	Exp4	Mean	SD
Km (uM)	3.51	3.08	3.17	3.03	3.20	0.21
Vmax (nmol/min/mg protein)	510.50	206.20	313.60	391.74	355.51	128.30
CLint (mL/min/mg protein)	145.52	66.88	99.06	129.15	110.15	34.67
	POR-H2					
	Exp1	Exp2	Exp3	Exp4	Mean	SD
Km (uM)	3.42	2.73	2.90	3.78	3.21	0.48
Vmax (nmol/min/mg protein)	381.59	174.10	306.70	615.95	369.59	185.30
CLint (mL/min/mg protein)	111.46	63.76	105.78	163.04	111.01	40.69
	POR-H3					
	Exp1	Exp2	Exp3	Exp4	Mean	SD
Km (uM)	2.32	3.08	2.60	2.94	2.74	0.34
Vmax (nmol/min/mg protein)	195.79	180.59	249.07	344.10	242.39	73.89
CLint (mL/min/mg protein)	84.24	58.61	95.85	117.11	88.95	24.38
	POR-H4					
	Exp1	Exp2	Exp3	Exp4	Mean	SD
Km (uM)	2.77	2.39	2.19	2.59	2.49	0.25
Vmax (nmol/min/mg protein)	247.92	133.82	287.56	307.80	244.27	77.72
CLint (mL/min/mg protein)	89.59	55.94	131.08	118.72	98.83	33.47

S12 Table. Effect of POR mutations on cytochrome c reduction by POR co-expressed with (A) CYP2B11 or (B) CYP2D15 in Sf9 cells. Shown are rates of microsomal cytochrome c reduction by POR-H1 and each POR variant (H2-H4) at fixed NADPH concentration. Microsomal samples from each of four independently generated protein preparations (Experiment # 1-4) were independently tested three times each. The activities shown below are the average of 3 independent incubations of each protein preparation. Other details are provided in the Materials and Methods section. SD – standard deviation

	nmole Cytochrome C reduced/min/mg protein					
CYP2B11	Exp1	Exp2	Exp3	Exp4	Mean	SD
PFEV	11.1	7.0	10.0	6.0	8.5	2.4
POR-H1	183.9	250.0	115.1	204.8	188.5	56.1
POR-H2	177.7	169.5	162.2	105.9	153.8	32.6
POR-H3	142.8	91.1	150.2	144.8	132.2	27.6
POR-H4	28.3	60.9	116.7	95.5	75.3	38.9
	nmole Cytochrome C reduced/min/mg protein					
CYP2D15	Exp1	Exp2	Exp3	Exp4	Mean	SD
PFEV	8.8	6.5	5.8	6.3	6.8	1.4
POR-H1	133.4	161.9	154.7	229.1	169.8	41.3
POR-H2	134.9	135.9	162.0	199.8	158.1	30.5
POR-H3	50.1	150.8	142.5	116.7	115.0	45.6
POR-H4	88.3	94.1	87.2	165.3	108.7	37.9

S13 Table. Results of an enzyme kinetic study evaluating the effect of POR mutations on CYP2B11 enzyme function when coexpressed in Sf9 cells. Shown are rates of resorufin formation at different benzyloxyresorufin concentrations by CYP2B11 coexpressed with POR-H1, POR-H2, POR-H3, POR-H4, or pFastBac1 empty vector (PFEV) negative control in Sf9 microsomes. Microsomal samples from each of four independently generated protein preparations (Experiment # 1-4) were independently tested three times each. The activities shown below are the average of 3 independent incubations of each protein preparation. Other details are provided in the Materials and Methods section. SD – standard deviation

PFEV	pmol resorufin/min/pmol P450					
uM BROD	Exp1	Exp2	Exp3	Exp4	Mean	SD
0	0.000	0.000	0.000	0.000	0.000	0.000
0.5	0.015	0.013	0.035	0.015	0.020	0.011
0.75	0.020	0.018	0.047	0.025	0.027	0.013
1	0.028	0.028	0.047	0.028	0.033	0.010
2.5	0.050	0.032	0.099	0.047	0.057	0.029
5	0.047	0.047	0.099	0.037	0.057	0.028
10	0.053	0.062	0.121	0.070	0.076	0.031
POR-H1	pmol resorufin/min/pmol P450					
uM BROD	Exp1	Exp2	Exp3	Exp4	Mean	SD
0	0.000	0.000	0.000	0.000	0.000	0.000
0.5	0.893	0.776	0.879	0.508	0.764	0.178
0.75	1.328	1.246	1.454	0.802	1.208	0.283
1	1.670	1.615	1.754	1.066	1.526	0.312
2.5	2.375	2.267	2.431	1.440	2.128	0.464
5	2.622	2.437	2.663	1.564	2.322	0.515
10	2.985	3.057	2.945	1.727	2.679	0.636
POR-H2	pmol resorufin/min/pmol P450					
uM BROD	Exp1	Exp2	Exp3	Exp4	Mean	SD
0	0.000	0.000	0.000	0.000	0.000	0.000
0.5	0.851	0.664	0.678	0.585	0.694	0.112
0.75	1.298	1.060	1.090	0.865	1.078	0.177
1	1.653	1.369	1.278	1.134	1.358	0.219
2.5	2.275	1.884	1.705	1.484	1.837	0.334
5	2.367	2.116	1.839	1.671	1.998	0.307
10	3.490	2.254	1.984	2.039	2.442	0.709
POR-H3	pmol resorufin/min/pmol P450					
uM BROD	Exp1	Exp2	Exp3	Exp4	Mean	SD

0	0.000	0.000	0.000	0.000	0.000	0.000
0.5	0.752	0.453	0.412	0.357	0.494	0.177
0.75	1.157	0.741	0.607	0.549	0.764	0.274
1	1.439	0.861	0.723	0.715	0.935	0.343
2.5	1.971	1.278	0.952	1.010	1.303	0.467
5	2.042	1.388	1.083	1.061	1.393	0.457
10	2.196	1.603	1.379	1.178	1.589	0.440
POR-H4	pmol resorufin/min/pmol P450					
uM BROD	Exp1	Exp2	Exp3	Exp4	Average	SD
0	0.000	0.000	0.000	0.000	0.000	0.000
0.5	0.118	0.249	0.381	0.262	0.252	0.108
0.75	0.200	0.430	0.587	0.363	0.395	0.160
1	0.219	0.518	0.718	0.449	0.476	0.206
2.5	0.357	0.643	0.976	0.652	0.657	0.253
5	0.368	0.658	1.050	0.696	0.693	0.279
10	0.431	0.654	1.298	0.818	0.800	0.367

S14 Table. Results of an enzyme kinetic study evaluating the effect of POR mutations on CYP2B11 enzyme function when coexpressed in Sf9 cells. Shown are rates of 4-hydroxypropofol formation at different propofol concentrations by CYP2B11 coexpressed with POR-H1, POR-H2, POR-H3, POR-H4, or pFastBac1 empty vector (PFEV) negative control in Sf9 microsomes. Microsomal samples from each of four independently generated protein preparations (Experiment # 1-4) were independently tested three times each. The activities shown below are the average of 3 independent incubations of each protein preparation. Other details are provided in the Materials and Methods section. SD – standard deviation

PFEV	pmol 4OH-Propofol/min/pmol P450					
uM Propofol	Exp1	Exp2	Exp3	Exp4	Mean	SD
0	0.00	0.00	0.00	0.00	0.00	0.00
2.5	0.00	0.00	0.00	0.00	0.00	0.00
5	0.00	0.18	0.16	0.17	0.13	0.08
10	0.10	0.38	0.27	0.32	0.27	0.12
25	0.18	0.49	0.76	0.63	0.52	0.25
50	0.35	0.62	0.88	0.75	0.65	0.23
75	0.41	0.66	0.96	0.81	0.71	0.24
100	0.44	0.81	0.93	0.87	0.76	0.22
POR-H1	pmol 4OH-Propofol/min/pmol P450					
uM Propofol	Exp1	Exp2	Exp3	Exp4	Mean	SD
0	0.00	0.00	0.00	0.00	0.00	0.00
2.5	4.05	4.55	5.47	3.66	4.44	0.78
5	5.93	5.97	7.13	4.38	5.85	1.13
10	7.25	7.63	8.55	5.39	7.21	1.33
25	10.03	9.25	11.44	6.49	9.31	2.08
50	12.18	11.52	14.27	7.54	11.38	2.82
75	13.39	12.27	16.97	8.81	12.86	3.36
100	15.06	13.33	15.52	9.82	13.43	2.59
POR-H2	pmol 4OH-Propofol/min/pmol P450					
uM Propofol	Exp1	Exp2	Exp3	Exp4	Mean	SD
0	0.00	0.00	0.00	0.00	0.00	0.00
2.5	3.24	4.19	3.77	3.32	3.63	0.44
5	5.64	5.47	5.14	4.11	5.09	0.69
10	6.95	7.73	7.00	5.63	6.83	0.87
25	9.77	9.64	7.97	6.23	8.40	1.66
50	11.31	11.36	10.14	8.20	10.25	1.48
75	13.50	12.92	11.21	8.81	11.61	2.11
100	13.24	12.98	11.67	9.34	11.81	1.78

POR-H3	pmol 4OH-Propofol/min/pmol P450					
uM Propofol	Exp1	Exp2	Exp3	Exp4	Mean	SD
0	0.00	0.00	0.00	0.00	0.00	0.00
2.5	3.21	2.99	1.63	2.08	2.48	0.75
5	4.68	4.02	2.30	2.68	3.42	1.12
10	6.50	5.34	3.13	3.51	4.62	1.58
25	8.51	6.76	3.91	4.65	5.96	2.09
50	9.99	8.03	4.79	4.91	6.93	2.53
75	11.36	8.64	5.15	5.39	7.64	2.95
100	11.95	9.14	5.40	6.38	8.22	2.95
POR-H4	pmol 4OH-Propofol/min/pmol P450					
uM Propofol	Exp1	Exp2	Exp3	Exp4	Average	SD
0	0.00	0.00	0.00	0.00	0.00	0.00
2.5	0.25	1.35	2.00	1.47	1.27	0.74
5	0.58	1.89	2.88	1.49	1.71	0.95
10	1.10	2.47	3.05	1.96	2.15	0.83
25	1.60	3.28	4.74	2.59	3.05	1.32
50	1.95	3.76	5.54	2.99	3.56	1.51
75	2.21	4.51	5.99	3.27	3.99	1.63
100	2.50	5.03	5.75	3.64	4.23	1.45

S15 Table. Results of an enzyme kinetic study evaluating the effect of POR mutations on CYP2B11 enzyme function when coexpressed in Sf9 cells. Shown are rates of 6-hydroxybupropion formation at different bupropion concentrations by CYP2B11 coexpressed with POR-H1, POR-H2, POR-H3, POR-H4, or pFastBac1 empty vector (PFEV) negative control in Sf9 microsomes. Microsomal samples from each of four independently generated protein preparations (Experiment # 1-4) were independently tested three times each. The activities shown below are the average of 3 independent incubations of each protein preparation. Other details are provided in the Materials and Methods section. SD – standard deviation

PFEV	pmol OH-Bupropion/min/pmol P450					
uM Bupropion	Exp1	Exp2	Exp3	Exp4	Mean	SD
0	0.00	0.00	0.00	0.00	0.00	0.00
5	0.00	0.00	0.00	0.00	0.00	0.00
10	0.00	0.00	0.06	0.06	0.03	0.03
25	0.00	0.00	0.15	0.09	0.06	0.07
50	0.06	0.09	0.17	0.11	0.11	0.05
100	0.18	0.13	0.31	0.26	0.22	0.08
250	0.34	0.21	0.42	0.36	0.33	0.09
500	0.47	0.32	0.57	0.61	0.49	0.13
POR-H1	pmol OH-Bupropion/min/pmol P450					
uM Bupropion	Exp1	Exp2	Exp3	Exp4	Average	SD
0	0.00	0.00	0.00	0.00	0.00	0.00
5	0.96	0.69	0.82	0.85	0.83	0.11
10	1.05	0.95	1.11	1.15	1.06	0.09
25	1.54	1.32	1.80	1.50	1.54	0.20
50	1.88	1.48	2.14	1.84	1.83	0.27
100	2.57	2.22	2.83	2.54	2.54	0.25
250	3.08	2.73	3.75	3.24	3.20	0.42
500	3.75	3.10	4.49	3.86	3.80	0.57
POR-H2	pmol OH-Bupropion/min/pmol P450					
uM Bupropion	Exp1	Exp2	Exp3	Exp4	Average	SD
0	0.00	0.00	0.00	0.00	0.00	0.00
5	0.65	0.57	0.59	0.75	0.64	0.08
10	0.83	0.86	0.72	1.07	0.87	0.15
25	1.37	1.18	1.17	1.55	1.32	0.18
50	1.90	1.36	1.47	1.98	1.68	0.31
100	2.31	1.98	1.94	2.42	2.16	0.24
250	3.00	2.63	2.44	3.40	2.87	0.42
500	3.49	2.67	2.97	4.01	3.29	0.59
POR-H3	pmol OH-Bupropion/min/pmol P450					
uM Bupropion	Exp1	Exp2	Exp3	Exp4	Average	SD

0	0.00	0.00	0.00	0.00	0.00	0.00
5	0.64	0.44	0.28	0.40	0.44	0.15
10	0.89	0.58	0.43	0.69	0.65	0.19
25	1.25	0.83	0.66	0.91	0.91	0.25
50	1.74	1.16	0.88	1.16	1.24	0.36
100	2.18	1.40	0.99	1.46	1.51	0.49
250	2.77	1.76	1.36	2.05	1.99	0.59
500	3.17	2.02	1.50	2.39	2.27	0.70
POR-H4	pmol OH-Bupropion/min/pmol P450					
uM Bupropion	Exp1	Exp2	Exp3	Exp4	Average	SD
0	0.00	0.00	0.00	0.00	0.00	0.00
5	0.00	0.18	0.39	0.31	0.22	0.17
10	0.19	0.22	0.60	0.51	0.38	0.20
25	0.31	0.38	0.74	0.72	0.54	0.23
50	0.45	0.48	1.01	0.86	0.70	0.28
100	0.62	0.66	1.27	1.22	0.94	0.35
250	0.89	0.88	1.68	1.58	1.26	0.43
500	1.10	1.11	1.97	2.01	1.55	0.51

S16 Table. Results of an enzyme kinetic study evaluating the effect of POR mutations on CYP2D15 enzyme function when coexpressed in Sf9 cells. Shown are rates of O-desmethyltramadol formation at different tramadol concentrations by CYP2D15 coexpressed with POR-H1, POR-H2, POR-H3, POR-H4, or pFastBac1 empty vector (PFEV) negative control in Sf9 microsomes. Microsomal samples from each of four independently generated protein preparations (Experiment # 1-4) were independently tested three times each. The activities shown below are the average of 3 independent incubations of each protein preparation. Other details are provided in the Materials and Methods section. SD – standard deviation

PFEV	pmoles O-desmethyltramadol/min/pmol P450					
uM Tramadol	Exp1	Exp2	Exp3	Exp4	Mean	SD
0	0.00	0.00	0.00	0.00	0.00	0.00
1	0.11	0.13	0.10	0.02	0.09	0.05
2	0.23	0.21	0.17	0.10	0.18	0.06
5	0.38	0.38	0.34	0.21	0.33	0.08
10	0.65	0.67	0.55	0.28	0.54	0.18
20	0.96	0.83	0.73	0.26	0.70	0.30
50	1.18	1.23	1.03	0.43	0.97	0.37
100	1.42	1.46	1.31	0.49	1.17	0.46
POR-H1	pmoles O-desmethyltramadol/min/pmol P450					
uM Tramadol	Exp1	Exp2	Exp3	Exp4	Mean	SD
0	0.00	0.00	0.00	0.00	0.00	0.00
1	1.27	0.85	1.95	1.90	1.49	0.53
2	2.05	1.47	3.19	2.85	2.39	0.78
5	4.08	2.43	6.05	5.03	4.40	1.53
10	5.93	4.16	8.35	6.61	6.26	1.73
20	7.76	5.16	10.55	5.92	7.35	2.40
50	9.75	6.53	13.10	10.39	9.94	2.70
100	10.29	6.99	13.98	11.44	10.67	2.90
POR-H2	pmoles O-desmethyltramadol/min/pmol P450					
uM Tramadol	Exp1	Exp2	Exp3	Exp4	Mean	SD
0	0.00	0.00	0.00	0.00	0.00	0.00
1	0.71	1.07	1.86	1.64	1.32	0.53
2	1.17	1.93	3.16	2.56	2.20	0.85
5	2.19	3.49	5.75	4.94	4.09	1.58
10	3.24	4.97	8.42	7.20	5.96	2.31
20	4.25	6.20	10.62	9.05	7.53	2.85
50	5.19	7.82	12.81	10.74	9.14	3.33
100	5.92	8.32	14.10	11.71	10.02	3.62

POR-H3	pmoles O-desmethyltramadol/min/pmol P450					
uM Tramadol	Exp1	Exp2	Exp3	Exp4	Mean	SD
0	0.00	0.00	0.00	0.00	0.00	0.00
1	0.58	1.59	1.58	1.16	1.23	0.48
2	0.56	2.62	2.93	1.93	2.01	1.05
5	1.69	4.69	5.49	3.37	3.81	1.66
10	2.41	6.19	7.05	4.43	5.02	2.05
20	3.03	7.66	8.22	5.48	6.10	2.36
50	3.80	8.73	10.05	6.59	7.29	2.73
100	4.46	9.28	10.41	6.88	7.76	2.64
POR-H4	pmoles O-desmethyltramadol/min/pmol P450					
uM Tramadol	Exp1	Exp2	Exp3	Exp4	Mean	SD
0	0.00	0.00	0.00	0.00	0.00	0.00
1	1.45	1.49	1.94	1.47	1.59	0.24
2	1.28	2.50	3.29	2.33	2.35	0.83
5	3.99	4.56	5.90	3.80	4.56	0.95
10	6.04	6.41	8.21	5.18	6.46	1.28
20	7.25	8.13	10.33	5.33	7.76	2.08
50	8.56	9.84	12.10	6.77	9.32	2.24
100	9.02	10.34	13.15	7.19	9.93	2.51

S17 Table. Results of an enzyme kinetic study evaluating the effect of POR mutations on CYP2D15 enzyme function when coexpressed in Sf9 cells. Shown are rates of dextrorphan formation at different dextromethorphan concentrations by CYP2D15 coexpressed with POR-H1, POR-H2, POR-H3, POR-H4, or pFastBac1 empty vector (PFEV) negative control in Sf9 microsomes. Microsomal samples from each of four independently generated protein preparations (Experiment # 1-4) were independently tested three times each. The activities shown below are the average of 3 independent incubations of each protein preparation. Other details are provided in the Materials and Methods section. SD – standard deviation

PFEV	pmol dextrorphan formed/min/pmol P450					
uM Dextromethorphan	Exp1	Exp2	Exp3	Exp4	Mean	SD
0	0.00	0.00	0.00	0.00	0.00	0.00
0.1	0.00	0.00	0.04	0.03	0.02	0.02
0.25	0.12	0.16	0.13	0.06	0.12	0.04
0.5	0.29	0.37	0.26	0.16	0.27	0.08
1	0.51	0.86	0.45	0.34	0.54	0.22
5	0.90	1.38	0.84	0.59	0.93	0.33
10	1.03	1.67	0.95	0.68	1.08	0.42
25	1.18	1.70	1.17	0.71	1.19	0.40
POR-H1	pmol dextrorphan formed/min/pmol P450					
uM Dextromethorphan	Exp1	Exp2	Exp3	Exp4	Mean	SD
0	0.00	0.00	0.00	0.00	0.00	0.00
0.1	0.44	0.42	0.97	0.71	0.64	0.26
0.25	1.26	1.20	2.11	1.64	1.55	0.42
0.5	2.20	1.86	3.23	2.57	2.46	0.58
1	3.58	2.91	4.83	3.85	3.79	0.80
5	4.73	4.06	6.63	5.19	5.15	1.09
10	5.45	4.41	6.96	5.68	5.62	1.05
25	5.48	4.74	7.41	5.98	5.90	1.13
POR-H2	pmol dextrorphan formed/min/pmol P450					
uM Dextromethorphan	Exp1	Exp2	Exp3	Exp4	Mean	SD
0	0.00	0.00	0.00	0.00	0.00	0.00
0.1	0.28	0.56	0.86	0.67	0.59	0.24
0.25	0.78	1.35	2.00	1.80	1.48	0.54
0.5	1.09	2.21	3.10	2.56	2.24	0.85
1	2.05	3.39	4.40	4.16	3.50	1.06
5	2.76	4.44	6.60	5.31	4.78	1.61
10	2.91	4.81	6.94	5.61	5.06	1.69
25	3.03	4.65	7.34	6.21	5.31	1.88

POR-H3	pmol dextrorphan formed/min/pmol P450					
uM Dextromethorphan	Exp1	Exp2	Exp3	Exp4	Mean	SD
0	0.00	0.00	0.00	0.00	0.00	0.00
0.1	0.25	0.69	0.66	0.38	0.49	0.21
0.25	0.64	1.49	1.33	1.18	1.16	0.37
0.5	0.97	2.40	2.41	1.73	1.88	0.69
1	1.58	4.25	3.61	3.03	3.12	1.14
5	2.23	6.16	4.81	3.90	4.27	1.65
10	2.23	6.37	5.13	4.00	4.43	1.76
25	2.46	6.68	5.68	4.24	4.76	1.83
POR-H4	pmol dextrorphan formed/min/pmol P450					
uM Dextromethorphan	Exp1	Exp2	Exp3	Exp4	Mean	SD
0	0.00	0.00	0.00	0.00	0.00	0.00
0.1	0.59	0.75	1.04	0.59	0.74	0.21
0.25	1.63	1.68	2.13	1.18	1.66	0.39
0.5	2.38	2.75	2.91	1.83	2.47	0.48
1	3.67	4.52	4.97	2.97	4.03	0.89
5	4.70	5.95	6.34	3.87	5.22	1.14
10	5.55	6.20	7.23	4.68	5.92	1.08
25	5.91	7.01	7.70	4.92	6.39	1.22

S18 Table. Results of an enzyme kinetic study evaluating the effect of POR mutations on CYP2B11 enzyme function when coexpressed in Sf9 cells. Shown are Michaelis-Menten parameters Km, Vmax and CLint (intrinsic clearance; Vmax / Km) for microsomal resorufin formation at different benzyloxyresorufin concentrations by CYP2B11 coexpressed with POR-H1, POR-H2, POR-H3, POR-H4, or pFastBac1 empty vector (PFEV) negative control in Sf9 microsomes. Enzyme kinetic parameters were derived by fitting a one-enzyme Michaelis-Menten model to measured enzyme activities and substrate concentrations using nonlinear regression. Microsomal samples from each of four independently generated protein preparations (Experiment # 1-4) were independently tested three times each. The parameters shown below are the average of 3 independent incubations of each protein preparation. Other details are provided in the Materials and Methods section. SD – standard deviation.

	PFEV					
	Exp1	Exp2	Exp3	Exp4	Mean	SD
Km (uM)	1.21	2.30	1.49	1.64	1.66	0.46
Vmax (pmol/min/pmol P450)	0.06	0.07	0.14	0.07	0.09	0.04
Clint (µL/min/pmol P450)	0.05	0.03	0.09	0.04	0.05	0.03
	POR-H1					
	Exp1	Exp2	Exp3	Exp4	Mean	SD
Km (uM)	1.10	1.27	0.99	1.01	1.09	0.13
Vmax (pmol/min/pmol P450)	3.30	3.32	3.26	1.92	2.95	0.69
Clint (µL/min/pmol P450)	2.99	2.61	3.30	1.91	2.70	0.60
	POR-H2					
	Exp1	Exp2	Exp3	Exp4	Mean	SD
Km (uM)	1.48	1.03	0.81	1.15	1.12	0.28
Vmax (pmol/min/pmol P450)	3.65	2.55	2.17	2.19	2.64	0.69
Clint (µL/min/pmol P450)	2.46	2.47	2.68	1.90	2.38	0.34
	POR-H3					
	Exp1	Exp2	Exp3	Exp4	Mean	SD
Km (uM)	0.82	1.13	1.14	0.99	1.02	0.15
Vmax (pmol/min/pmol P450)	2.44	1.77	1.44	1.31	1.74	0.50
Clint (µL/min/pmol P450)	2.96	1.57	1.26	1.32	1.78	0.80
	POR-H4					
	Exp1	Exp2	Exp3	Exp4	Mean	SD
Km (uM)	1.17	0.59	1.07	1.07	0.98	0.26
Vmax (pmol/min/pmol P450)	0.48	0.74	1.38	0.89	0.87	0.38
Clint (µL/min/pmol P450)	0.41	1.25	1.28	0.83	0.94	0.41

S19 Table. Results of an enzyme kinetic study evaluating the effect of POR mutations on CYP2B11 enzyme function when coexpressed in Sf9 cells. Shown are Michaelis-Menten parameters Km, Vmax and CLint (intrinsic clearance; Vmax / Km) for microsomal 4-hydroxypropofol formation at different propofol concentrations by CYP2B11 coexpressed with POR-H1, POR-H2, POR-H3, POR-H4, or pFastBac1 empty vector (PFEV) negative control in Sf9 microsomes. Enzyme kinetic parameters were derived by fitting a one-enzyme Michaelis-Menten model to measured enzyme activities and substrate concentrations using nonlinear regression. Microsomal samples from each of four independently generated protein preparations (Experiment # 1-4) were independently tested three times each. The parameters shown below are the average of 3 independent incubations of each protein preparation. Other details are provided in the Materials and Methods section. SD – standard deviation.

	PFEV					
	Exp1	Exp2	Exp3	Exp4	Mean	SD
Km (uM)	21.18	20.01	25.68	23.40	22.57	2.51
Vmax (pmol/min/pmol P450)	0.58	0.90	1.27	1.09	0.96	0.30
Clint (µL/min/pmol P450)	0.03	0.05	0.05	0.05	0.04	0.01
	POR-H1					
	Exp1	Exp2	Exp3	Exp4	Mean	SD
Km (uM)	9.58	6.55	7.93	5.90	7.49	1.63
Vmax (pmol/min/pmol P450)	15.20	13.22	16.94	9.21	13.64	3.32
Clint (µL/min/pmol P450)	1.59	2.02	2.14	1.56	1.83	0.30
	POR-H2					
	Exp1	Exp2	Exp3	Exp4	Mean	SD
Km (uM)	9.53	7.36	6.91	6.43	7.56	1.37
Vmax (pmol/min/pmol P450)	14.30	13.54	11.81	9.31	12.24	2.21
Clint (µL/min/pmol P450)	1.50	1.84	1.71	1.45	1.62	0.18
	POR-H3					
	Exp1	Exp2	Exp3	Exp4	Mean	SD
Km (uM)	8.91	6.84	7.56	6.39	7.43	1.10
Vmax (pmol/min/pmol P450)	12.40	9.30	5.58	6.04	8.33	3.18
Clint (µL/min/pmol P450)	1.39	1.36	0.74	0.94	1.11	0.32
	POR-H4					
	Exp1	Exp2	Exp3	Exp4	Mean	SD
Km (uM)	19.02	9.69	7.09	8.74	11.14	5.37
Vmax (pmol/min/pmol P450)	2.84	5.00	6.25	4.37	4.62	1.42
Clint (µL/min/pmol P450)	0.15	0.52	0.88	0.50	0.51	0.30

S20 Table. Results of an enzyme kinetic study evaluating the effect of POR mutations on CYP2B11 enzyme function when coexpressed in Sf9 cells. Shown are Michaelis-Menten parameters Km, Vmax and CLint (intrinsic clearance; Vmax / Km) for microsomal 6-hydroxybupropion formation at different bupropion concentrations by CYP2B11 coexpressed with POR-H1, POR-H2, POR-H3, POR-H4, or pFastBac1 empty vector (PFEV) negative control in Sf9 microsomes. Enzyme kinetic parameters were derived by fitting a one-enzyme Michaelis-Menten model to measured enzyme activities and substrate concentrations using nonlinear regression. Microsomal samples from each of four independently generated protein preparations (Experiment # 1-4) were independently tested three times each. The parameters shown below are the average of 3 independent incubations of each protein preparation. Other details are provided in the Materials and Methods section. SD – standard deviation.

	PFEV					
	Exp1	Exp2	Exp3	Exp4	Mean	SD
Km (uM)	488.31	357.33	131.57	383.42	340.16	150.14
Vmax (pmol/min/pmol P450)	0.95	0.54	0.69	1.04	0.80	0.23
Clint (µL/min/pmol P450)	0.00	0.00	0.01	0.00	0.00	0.00
	POR-H1					
	Exp1	Exp2	Exp3	Exp4	Mean	SD
Km (uM)	34.71	36.92	45.87	40.52	39.50	4.87
Vmax (pmol/min/pmol P450)	3.67	3.15	4.57	3.87	3.82	0.59
Clint (µL/min/pmol P450)	0.11	0.09	0.10	0.10	0.10	0.01
	POR-H2					
	Exp1	Exp2	Exp3	Exp4	Mean	SD
Km (uM)	41.54	36.68	43.05	45.94	41.80	3.87
Vmax (pmol/min/pmol P450)	3.58	2.85	3.00	4.08	3.38	0.57
Clint (µL/min/pmol P450)	0.09	0.08	0.07	0.09	0.08	0.01
	POR-H3					
	Exp1	Exp2	Exp3	Exp4	Mean	SD
Km (uM)	37.58	33.27	33.79	46.83	37.87	6.28
Vmax (pmol/min/pmol P450)	3.23	2.02	1.52	2.45	2.31	0.72
Clint (µL/min/pmol P450)	0.09	0.06	0.05	0.05	0.06	0.02
	POR-H4					
	Exp1	Exp2	Exp3	Exp4	Mean	SD
Km (uM)	92.04	63.45	40.51	56.56	63.14	21.53
Vmax (pmol/min/pmol P450)	1.26	1.17	1.99	2.07	1.62	0.47
Clint (µL/min/pmol P450)	0.01	0.02	0.05	0.04	0.03	0.02

S21 Table. Results of an enzyme kinetic study evaluating the effect of POR mutations on CYP2D15 enzyme function when coexpressed in Sf9 cells. Shown are Michaelis-Menten parameters Km, Vmax and CLint (intrinsic clearance; Vmax / Km) for microsomal O-desmethyltramadol formation at different tramadol concentrations by CYP2D15 coexpressed with POR-H1, POR-H2, POR-H3, POR-H4, or pFastBac1 empty vector (PFEV) negative control in Sf9 microsomes. Enzyme kinetic parameters were derived by fitting a one-enzyme Michaelis-Menten model to measured enzyme activities and substrate concentrations using nonlinear regression. Microsomal samples from each of four independently generated protein preparations (Experiment # 1-4) were independently tested three times each. The parameters shown below are the average of 3 independent incubations of each protein preparation. Other details are provided in the Materials and Methods section. SD – standard deviation.

	PFEV					
	Exp1	Exp2	Exp3	Exp4	Mean	SD
Km (uM)	14.32	17.08	18.73	9.99	15.03	3.82
Vmax (pmol/min/pmol P450)	1.59	1.67	1.50	0.51	1.32	0.54
Clint (µL/min/pmol P450)	0.11	0.10	0.08	0.05	0.09	0.03
	POR-H1					
	Exp1	Exp2	Exp3	Exp4	Mean	SD
Km (uM)	8.89	9.26	7.65	8.21	8.50	0.72
Vmax (pmol/min/pmol P450)	11.30	7.67	14.96	11.58	11.38	2.98
Clint (µL/min/pmol P450)	1.27	0.83	1.96	1.41	1.37	0.47
	POR-H2					
	Exp1	Exp2	Exp3	Exp4	Mean	SD
Km (uM)	9.48	7.93	7.89	7.51	8.20	0.87
Vmax (pmol/min/pmol P450)	6.33	8.95	15.00	12.48	10.69	3.82
Clint (µL/min/pmol P450)	0.67	1.13	1.90	1.66	1.34	0.55
	POR-H3					
	Exp1	Exp2	Exp3	Exp4	Mean	SD
Km (uM)	10.37	5.44	5.46	5.90	6.79	2.39
Vmax (pmol/min/pmol P450)	4.76	9.72	10.94	7.26	8.17	2.74
Clint (µL/min/pmol P450)	0.46	1.79	2.00	1.23	1.37	0.69
	POR-H4					
	Exp1	Exp2	Exp3	Exp4	Mean	SD
Km (uM)	7.43	7.13	6.72	4.49	6.44	1.33
Vmax (pmol/min/pmol P450)	9.85	11.11	13.86	7.26	10.52	2.75
Clint (µL/min/pmol P450)	1.33	1.56	2.06	1.62	1.64	0.31

Table S22. Results of an enzyme kinetic study evaluating the effect of POR mutations on CYP2D15 enzyme function when coexpressed in Sf9 cells. Shown are Michaelis-Menten parameters Km, Vmax and CLint (intrinsic clearance; Vmax / Km) for microsomal dextrorphan formation at different dextromethorphan concentrations by CYP2D15 coexpressed with POR-H1, POR-H2, POR-H3, POR-H4, or pFastBac1 empty vector (PFEV) negative control in Sf9 microsomes. Enzyme kinetic parameters were derived by fitting a one-enzyme Michaelis-Menten model to measured enzyme activities and substrate concentrations using nonlinear regression. Microsomal samples from each of four independently generated protein preparations (Experiment # 1-4) were independently tested three times each. The parameters shown below are the average of 3 independent incubations of each protein preparation. Other details are provided in the Materials and Methods section. SD – standard deviation.

	PFEV					
	Exp1	Exp2	Exp3	Exp4	Mean	SD
Km (uM)	1.71	1.57	1.93	1.93	1.79	0.18
Vmax (pmol/min/pmol P450)	1.24	1.86	1.20	1.20	1.37	0.32
Clint (µL/min/pmol P450)	0.72	1.18	0.62	0.62	0.79	0.27
	POR-H1					
	Exp1	Exp2	Exp3	Exp4	Mean	SD
Km (uM)	0.76	0.75	0.63	0.65	0.70	0.07
Vmax (pmol/min/pmol P450)	5.70	4.79	7.51	6.05	6.01	1.13
Clint (µL/min/pmol P450)	7.50	6.39	12.01	9.26	8.79	2.45
	POR-H2					
	Exp1	Exp2	Exp3	Exp4	Mean	SD
Km (uM)	0.74	0.59	0.71	0.62	0.66	0.07
Vmax (pmol/min/pmol P450)	3.15	4.96	7.51	6.15	5.44	1.85
Clint (µL/min/pmol P450)	4.25	8.42	10.58	9.96	8.30	2.85
	POR-H3					
	Exp1	Exp2	Exp3	Exp4	Mean	SD
Km (uM)	0.70	0.81	0.68	0.64	0.71	0.07
Vmax (pmol/min/pmol P450)	2.50	6.99	5.64	4.36	4.87	1.91
Clint (µL/min/pmol P450)	3.54	8.65	8.32	6.85	6.84	2.33
	POR-H4					
	Exp1	Exp2	Exp3	Exp4	Mean	SD
Km (uM)	0.70	0.70	0.68	0.79	0.72	0.05
Vmax (pmol/min/pmol P450)	5.86	6.94	7.69	4.93	6.35	1.21
Clint (µL/min/pmol P450)	8.41	9.95	11.24	6.27	8.97	2.14
