# Supplementary material for: Pharmacogenomics of poor drug metabolism in greyhounds: Canine P450 oxidoreductase genetic variation, breed heterogeneity, and functional characterization
Source: PLoS One. 2024 Feb 1;19(2):e0297191. doi: 10.1371/journal.pone.0297191 (PMC10833530; doi:10.1371/journal.pone.0297191)
Supplement: S1 Data — (DOCX) [file pone.0297191.s010.docx]

**Raw data underlying all mean and standard deviation values presented in the manuscript**

**S5 Fig. POR protein immunoblots of microsomes from Sf9 cells expressing wild-type POR (H1) or each POR variant (H2, H3 or H4).**

**S6 Fig. CYP2B11 and POR protein immunoblots of microsomes from Sf9 cells coexpressing CYP2B11 with wild-type POR (H1) or each POR variant (H2, H3 or H4).**

**S7 Fig. CYP2D15 and POR protein immunoblots of microsomes from Sf9 cells coexpressing CYP2D15 with wild-type POR (H1) or each POR variant (H2, H3 or H4).**

**S5 Table. Results of quantitation of POR protein immunoblots (shown in S3 Fig) of microsomes from Sf9 cells expressing wild-type POR (H1) or each POR variant (H2, H3 or H4).**

**S6 Table. Results of quantitation of POR and CYP2B11 protein immunoblots (shown in S4 Fig) of microsomes from Sf9 cells coexpressing CYP2B11 with wild-type POR (H1) or each POR variant (H2, H3 or H4).**

**S7 Table. Results of quantitation of POR and CYP2D15 protein immunoblots (shown in S5 Fig) of microsomes from Sf9 cells coexpressing CYP2D15 with wild-type POR (H1) or each POR variant (H2, H3 or H4).**

**S8 Table. Rates of microsomal cytochrome c reduction by POR-H1 and each POR variant (H2-H4) when cytochrome c concentration is varied and NADPH concentration is fixed.**

**S9 Table. Michaelis-Menten parameters Km, Vmax and CLint (intrinsic clearance; Vmax / Km) for microsomal cytochrome c reduction by POR-H1 and each POR variant (H2-H4) when cytochrome c concentration is varied and NADPH concentration is fixed.**

**S10 Table. Rates of microsomal cytochrome c reduction by POR-H1 and each POR variant (H2-H4) when NADPH concentration is varied and cytochrome c concentration is fixed.**

**S11 Table. Michaelis-Menten parameters Km, Vmax and CLint (intrinsic clearance; Vmax / Km) for microsomal cytochrome c reduction by POR-H1 and each POR variant (H2-H4) when NADPH concentration is varied and cytochrome c concentration is fixed.**

**S12 Table. Rates of microsomal cytochrome c reduction by POR-H1 and each POR variant (H2-H4) co-expressed with CYP2B11 or CYP2D15.**

**S13 Table. Rates of resorufin formation at different benzyloxyresorufin concentrations by CYP2B11 coexpressed with POR-H1, POR-H2, POR-H3, POR-H4, or pFastBac1 empty vector (PFEV) negative control in Sf9 microsomes.**

**S14 Table. Rates of 4-hydroxypropofol formation at different propofol concentrations by CYP2B11 coexpressed with POR-H1, POR-H2, POR-H3, POR-H4, or pFastBac1 empty vector (PFEV) negative control in Sf9 microsomes.**

**S15 Table. Rates of 6-hydroxybupropion formation at different bupropion concentrations by CYP2B11 coexpressed with POR-H1, POR-H2, POR-H3, POR-H4, or pFastBac1 empty vector (PFEV) negative control in Sf9 microsomes.**

**S16 Table. Rates of O-desmethyltramadol formation at different tramadol concentrations by CYP2D15 coexpressed with POR-H1, POR-H2, POR-H3, POR-H4, or pFastBac1 empty vector (PFEV) negative control in Sf9 microsomes.**

**S17 Table. Rates of dextrorphan formation at different dextromethorphan concentrations by CYP2D15 coexpressed with POR-H1, POR-H2, POR-H3, POR-H4, or pFastBac1 empty vector (PFEV) negative control in Sf9 microsomes.**

**S18 Table. Michaelis-Menten parameters Km, Vmax and CLint (intrinsic clearance; Vmax / Km) for microsomal resorufin formation at different benzyloxyresorufin concentrations by CYP2B11 coexpressed with POR-H1, POR-H2, POR-H3, POR-H4, or pFastBac1 empty vector (PFEV) negative control in Sf9 microsomes.**

**S19 Table. Michaelis-Menten parameters Km, Vmax and CLint (intrinsic clearance; Vmax / Km) for microsomal 4-hydroxypropofol formation at different propofol concentrations by CYP2B11 coexpressed with POR-H1, POR-H2, POR-H3, POR-H4, or pFastBac1 empty vector (PFEV) negative control in Sf9 microsomes.**

**S20 Table. Michaelis-Menten parameters Km, Vmax and CLint (intrinsic clearance; Vmax / Km) for microsomal 6-hydroxybupropion formation at different bupropion concentrations by CYP2B11 coexpressed with POR-H1, POR-H2, POR-H3, POR-H4, or pFastBac1 empty vector (PFEV) negative control in Sf9 microsomes.**

**S21 Table. Michaelis-Menten parameters Km, Vmax and CLint (intrinsic clearance; Vmax / Km) for microsomal O-desmethyltramadol formation at different tramadol concentrations by CYP2D15 coexpressed with POR-H1, POR-H2, POR-H3, POR-H4, or pFastBac1 empty vector (PFEV) negative control in Sf9 microsomes.**

**S5 Fig.** POR protein immunoblots of microsomes from Sf9 cells expressing wild-type POR (H1) or each POR variant (H2, H3 or H4). Images of the same blots stained with Coomassie for total protein content are also shown. Tables on the left identify the samples loaded into each lane on the corresponding blot. Microsomal samples from each of four independently generated protein preparations (Experiment# 1-4) were blotted three times (Blot replicate # 1-3). β-glucuronidase (GUS) was used as the negative control and 1 or 3 ug of POR-H1 Sf9 microsomes (Std_1_ug; Std_3_ug) were used as the positive control. Other details are provided in the Materials and Methods section.

**S6 Fig.** CYP2B11 and POR protein immunoblots of microsomes from Sf9 cells coexpressing CYP2B11 with wild-type POR (H1) or each POR variant (H2, H3 or H4). Images of the same blots stained with Coomassie for total protein content are also shown. Tables to the left identify the samples loaded into each lane on the corresponding blot. Microsomal samples from each of four independently generated protein preparations (Experiment# 1-4) were blotted three times (Blot replicate # 1-3). Based on the significant size difference between the POR and CYP proteins, each blot was divided in half such that the top half contained POR (77 kDa) and the bottom half contained CYP2B11 (54 kDa). Each half was then incubated separately in the corresponding primary antibody, then together in the same secondary antibody, before chemiluminescence imaging. The reassembled top and botton halves are shown in the Coomassie stained blot image. pFastBac empty vector (PFEV) was used as the negative control and 1 or 3 ug of POR-H1 Sf9 microsomes (Std_1_ug; Std_3_ug) were used as positive controls. Other details are provided in the Materials and Methods section.

**S7 Fig.** CYP2D15 and POR protein immunoblots of microsomes from Sf9 cells coexpressing CYP2D15 with wild-type POR (H1) or each POR variant (H2, H3 or H4). Tables to the left identify the samples loaded into each lane on the corresponding blot. Microsomal samples from each of four independently generated protein preparations (Experiment# 1-4) were blotted three times (Blot replicate # 1-3). Based on the significant size difference between the POR and CYP proteins, each blot was divided in half such that the top half contained POR (77 kDa) and the bottom half contained CYP2D15 (54 kDa). Each half was then incubated separately in the corresponding primary antibody, then together in the same secondary antibody, before chemiluminescence imaging. The top and botton halves are shown in the Coomassie stained blot image. pFastBac empty vector (PFEV) was used as a POR negative control and microsomes from uninfected Sf9 cells were used as a POR and CYP2D15 negative control. Pooled dog liver microsomes (pDLMs) were used as a POR and CYP2D15 positive control. Other details are provided in the Materials and Methods section. In the CYP2D15 blots, note a prominent band that is faster migrating (about 10 kDa smaller) than the 54 kDa CYP2D15 band. This band is also found in uninfected Sf9 cells, but absent from pDLMs suggesting that it is a crossreactive background protein expressed in Sf9 cells.

**S5 Table.**  Results of quantitation of POR protein immunoblots (shown in S3 Fig) of microsomes from Sf9 cells expressing wild-type POR (H1) or each POR variant (H2, H3 or H4). Microsomal samples from each of four independently generated protein preparations (Experiment # 1-4) were blotted three times. The results below are the average of the total protein normalized band intensity for the 3 replicate blots in each protein preparation. Other details are provided in the Materials and Methods section. SD – standard deviation CV% - coefficient of variation as a percentage.

|  | POR protein band pixel volume normalized to total protein stained lane pixel volume (ratio) | | | | | | |
| --- | --- | --- | --- | --- | --- | --- | --- |
|  | Experiment # | | | |  |  |  |
| POR variant | Exp1 | Exp2 | Exp3 | Exp4 | Mean | SD | CV% |
| H1 | 218 | 222 | 213 | 249 | 225 | 16 | 7 |
| H2 | 203 | 197 | 212 | 245 | 214 | 21 | 10 |
| H3 | 211 | 195 | 211 | 239 | 214 | 18 | 9 |
| H4 | 229 | 214 | 194 | 255 | 223 | 26 | 12 |

**S6 Table.**  Results of quantitation of POR and CYP2B11 protein immunoblots (shown in S4 Fig) of microsomes from Sf9 cells coexpressing CYP2B11 with wild-type POR (H1) or each POR variant (H2, H3 or H4). Microsomal samples from each of four independently generated protein preparations (Experiment # 1-4) were blotted three times. The results below are the average of the total protein normalized band intensity for the 3 replicate blots in each protein preparation. Other details are provided in the Materials and Methods section. SD – standard deviation CV% - coefficient of variation.

|  | POR protein band pixel volume normalized to total protein stained lane pixel volume (ratio) | | | | | | |
| --- | --- | --- | --- | --- | --- | --- | --- |
|  | Experiment # | | | |  |  |  |
| POR variant | Exp1 | Exp2 | Exp3 | Exp4 | Mean | SD | CV% |
| PFEV (vector control) | 0 | 0 | 0 | 0 | 0 | 0 | 0 |
| H1 | 386 | 394 | 337 | 292 | 352 | 48 | 14 |
| H2 | 362 | 416 | 303 | 280 | 340 | 61 | 18 |
| H3 | 382 | 393 | 277 | 271 | 331 | 66 | 20 |
| H4 | 385 | 342 | 282 | 234 | 311 | 66 | 21 |
|  | | | | | | | |
|  | CYP2B11 protein band pixel volume normalized to total protein stained lane pixel volume (ratio) | | | | | | |
|  | Experiment # | | | |  |  |  |
| POR variant | Exp1 | Exp2 | Exp3 | Exp4 | Mean | SD | CV% |
| PFEV (vector control) | 442 | 454 | 369 | 424 | 422 | 38 | 9 |
| H1 | 481 | 395 | 345 | 345 | 392 | 64 | 16 |
| H2 | 416 | 463 | 366 | 387 | 408 | 42 | 10 |
| H3 | 488 | 461 | 353 | 365 | 417 | 68 | 16 |
| H4 | 435 | 435 | 377 | 330 | 394 | 51 | 13 |

**S7 Table.**  Results of quantitation of POR and CYP2D15 protein immunoblots (shown in S5 Fig) of microsomes from Sf9 cells coexpressing CYP2D15 with wild-type POR (H1) or each POR variant (H2, H3 or H4). Microsomal samples from each of four independently generated protein preparations (Experiment # 1-4) were blotted three times. The results below are the average of the total protein normalized band intensity for the 3 replicate blots in each protein preparation. Other details are provided in the Materials and Methods section. SD – standard deviation CV% - coefficient of variation.

|  | POR protein band volume normalized to total protein stained lane volume (ratio) | | | | | | |
| --- | --- | --- | --- | --- | --- | --- | --- |
|  | Experiment # | | | |  |  |  |
| POR variant | Exp1 | Exp2 | Exp3 | Exp4 | Mean | SD | CV% |
| PFEV (vector control) | 0 | 0 | 0 | 0 | 0 | 0 | 0 |
| H1 | 218 | 242 | 241 | 199 | 225 | 21 | 9 |
| H2 | 215 | 236 | 234 | 208 | 223 | 14 | 6 |
| H3 | 214 | 155 | 212 | 221 | 200 | 31 | 15 |
| H4 | 216 | 151 | 232 | 179 | 195 | 37 | 19 |
|  | | | | | | | |
|  | CYP2B11 protein band volume normalized to total protein stained lane volume (ratio) | | | | | | |
|  | Experiment # | | | |  |  |  |
| POR variant | Exp1 | Exp2 | Exp3 | Exp4 | Mean | SD | CV% |
| PFEV (vector control) | 196 | 217 | 169 | 153 | 184 | 29 | 16 |
| H1 | 214 | 242 | 219 | 190 | 217 | 21 | 10 |
| H2 | 216 | 226 | 222 | 200 | 216 | 11 | 5 |
| H3 | 233 | 219 | 178 | 222 | 213 | 24 | 11 |
| H4 | 237 | 158 | 222 | 182 | 200 | 36 | 18 |

**S8 Table.**  Results of an enzyme kinetic study evaluating the effect of POR mutations on cytochrome c reduction by POR expressed in Sf9 cells. Shown are rates of microsomal cytochrome c reduction by POR-H1 and each POR variant (H2-H4) when cytochrome c concentration is varied and NADPH concentration is fixed. Microsomal samples from each of four independently generated protein preparations (Experiment # 1-4) were independently tested three times each. The activities shown below are the average of 3 independent incubations of each protein preparation. Other details are provided in the Materials and Methods section. SD – standard deviation

| POR-H1 | nmole Cytochrome C reduced/min/mg protein | | | | | |
| --- | --- | --- | --- | --- | --- | --- |
| uM Cytochrome C | Exp1 | Exp2 | Exp3 | Exp4 | Mean | SD |
| 0 | 0.0 | 0.0 | 0.0 | 0.0 | 0.0 | 0.0 |
| 2.5 | 90.6 | 65.6 | 69.3 | 76.7 | 75.6 | 11.0 |
| 5 | 178.1 | 112.2 | 136.1 | 139.8 | 141.5 | 27.2 |
| 10 | 314.8 | 185.6 | 226.5 | 238.4 | 241.3 | 54.0 |
| 15 | 408.1 | 233.1 | 284.8 | 297.7 | 305.9 | 73.6 |
| 30 | 588.4 | 308.9 | 392.0 | 400.2 | 422.4 | 118.1 |
| 50 | 711.5 | 374.8 | 451.1 | 465.6 | 500.8 | 146.0 |
| 75 | 725.8 | 379.0 | 454.8 | 511.9 | 517.9 | 148.9 |
| 100 | 783.8 | 411.4 | 507.4 | 523.5 | 556.5 | 159.4 |
|  |  |  |  |  |  |  |
| POR-H2 | nmole Cytochrome C reduced/min/mg protein | | | | | |
| uM Cytochrome C | Exp1 | Exp2 | Exp3 | Exp4 | Mean | SD |
| 0 | 0.0 | 0.0 | 0.0 | 0.0 | 0.0 | 0.0 |
| 2.5 | 82.0 | 45.0 | 58.0 | 93.1 | 69.5 | 21.9 |
| 5 | 149.2 | 76.8 | 112.1 | 161.4 | 124.9 | 38.3 |
| 10 | 246.8 | 120.2 | 188.6 | 279.6 | 208.8 | 70.0 |
| 15 | 329.7 | 148.7 | 222.5 | 358.8 | 264.9 | 97.1 |
| 30 | 438.0 | 199.2 | 304.4 | 476.0 | 354.4 | 127.0 |
| 50 | 517.3 | 210.7 | 364.4 | 551.8 | 411.1 | 156.5 |
| 75 | 566.3 | 248.4 | 372.0 | 584.7 | 442.8 | 161.4 |
| 100 | 568.5 | 255.1 | 376.5 | 626.8 | 456.7 | 171.8 |
|  |  |  |  |  |  |  |
| POR-H3 | nmole Cytochrome C reduced/min/mg protein | | | | | |
| uM Cytochrome C | Exp1 | Exp2 | Exp3 | Exp4 | Mean | SD |
| 0 | 0.0 | 0.0 | 0.0 | 0.0 | 0.0 | 0.0 |
| 2.5 | 55.1 | 44.1 | 61.7 | 72.4 | 58.3 | 11.8 |
| 5 | 104.8 | 78.8 | 115.3 | 135.9 | 108.7 | 23.7 |
| 10 | 172.7 | 132.1 | 201.8 | 210.9 | 179.4 | 35.4 |
| 15 | 211.8 | 159.6 | 243.4 | 278.6 | 223.4 | 50.5 |
| 30 | 296.0 | 221.2 | 329.3 | 373.9 | 305.1 | 64.4 |
| 50 | 320.8 | 253.9 | 373.5 | 434.9 | 345.8 | 77.0 |
| 75 | 350.8 | 269.7 | 399.6 | 462.1 | 370.5 | 81.2 |
| 100 | 357.3 | 295.5 | 412.0 | 507.5 | 393.1 | 89.9 |
|  |  |  |  |  |  |  |
| POR-H4 | nmole Cytochrome C reduced/min/mg protein | | | | | |
| uM Cytochrome C | Exp1 | Exp2 | Exp3 | Exp4 | Mean | SD |
| 0 | 0.0 | 0.0 | 0.0 | 0.0 | 0.0 | 0.0 |
| 2.5 | 56.7 | 37.4 | 47.7 | 74.9 | 54.2 | 15.9 |
| 5 | 117.4 | 61.4 | 88.7 | 132.9 | 100.1 | 31.6 |
| 10 | 247.6 | 102.8 | 151.9 | 229.5 | 183.0 | 67.6 |
| 15 | 310.5 | 129.1 | 192.9 | 299.4 | 233.0 | 87.2 |
| 30 | 389.0 | 173.5 | 266.7 | 414.6 | 311.0 | 112.1 |
| 50 | 470.1 | 203.6 | 293.6 | 474.9 | 360.6 | 134.4 |
| 75 | 519.9 | 217.5 | 313.0 | 529.1 | 394.9 | 154.7 |
| 100 | 519.9 | 237.0 | 315.9 | 546.2 | 404.8 | 152.0 |

**S9 Table.**  Results of an enzyme kinetic study evaluating the effect of POR mutations on cytochrome c reduction by POR expressed in Sf9 cells. Shown are Michaelis-Menten parameters Km, Vmax and CLint (intrinsic clearance; Vmax / Km) for microsomal cytochrome c reduction by POR-H1 and each POR variant (H2-H4) when cytochrome c concentration is varied and NADPH concentration is fixed. Enzyme kinetic parameters were derived by fitting a one-enzyme Michaelis-Menten model to measured enzyme activities and substrate concentrations (data in Table S8) using nonlinear regression. Microsomal samples from each of four independently generated protein preparations (Experiment # 1-4) were independently tested three times each. The parameters shown below are the average of 3 independent incubations of each protein preparation. Other details are provided in the Materials and Methods section. SD – standard deviation

|  | POR-H1 | | | | | |
| --- | --- | --- | --- | --- | --- | --- |
|  | Exp1 | Exp2 | Exp3 | Exp4 | Mean | SD |
| Km (uM) | 19.7 | 15.5 | 15.5 | 16.2 | 16.7 | 2.0 |
| Vmax (nmol/min/mg protein) | 947.1 | 472.5 | 577.4 | 616.1 | 653.3 | 205.1 |
| CLint (mL/min/mg protein) | 48.1 | 30.5 | 37.2 | 37.9 | 38.5 | 7.3 |
|  |  |  |  |  |  |  |
|  | POR-H2 | | | | | |
|  | Exp1 | Exp2 | Exp3 | Exp4 | Mean | SD |
| Km (uM) | 16.9 | 14.0 | 14.2 | 14.2 | 14.8 | 1.4 |
| Vmax (nmol/min/mg protein) | 682.9 | 287.4 | 444.6 | 684.9 | 524.9 | 194.4 |
| CLint (mL/min/mg protein) | 40.3 | 20.5 | 31.3 | 48.2 | 35.1 | 11.9 |
|  |  |  |  |  |  |  |
|  | POR-H3 | | | | | |
|  | Exp1 | Exp2 | Exp3 | Exp4 | Mean | SD |
| Km (uM) | 14.1 | 16.1 | 14.4 | 16.9 | 15.4 | 1.3 |
| Vmax (nmol/min/mg protein) | 415.0 | 336.4 | 477.7 | 581.8 | 452.7 | 103.7 |
| CLint (mL/min/mg protein) | 29.4 | 20.9 | 33.1 | 34.4 | 29.5 | 6.1 |
|  |  |  |  |  |  |  |
|  | POR-H4 | | | | | |
|  | Exp1 | Exp2 | Exp3 | Exp4 | Mean | SD |
| Km (uM) | 17.1 | 16.6 | 14.3 | 18.2 | 16.5 | 1.6 |
| Vmax (nmol/min/mg protein) | 624.9 | 271.4 | 372.8 | 652.3 | 480.3 | 187.7 |
| CLint (mL/min/mg protein) | 36.6 | 16.3 | 26.1 | 35.9 | 28.7 | 9.6 |

**S10 Table.**  Results of an enzyme kinetic study evaluating the effect of POR mutations on cytochrome c reduction by POR expressed in Sf9 cells. Shown are rates of microsomal cytochrome c reduction by POR-H1 and each POR variant (H2-H4) when NADPH concentration is varied and cytochrome c concentration is fixed. Microsomal samples from each of four independently generated protein preparations (Experiment # 1-4) were independently tested three times each. The activities shown below are the average of 3 independent incubations of each protein preparation. Other details are provided in the Materials and Methods section. SD – standard deviation

| POR-H1 | nmole Cytochrome C reduced/min/mg protein | | | | | |
| --- | --- | --- | --- | --- | --- | --- |
| uM NADPH | Exp1 | Exp2 | Exp3 | Exp4 | Mean | SD |
| 0 | 0.0 | 0.0 | 0.0 | 0.0 | 0.0 | 0.0 |
| 1.5625 | 149.5 | 77.5 | 116.1 | 141.8 | 121.2 | 32.4 |
| 3.125 | 255.1 | 104.6 | 152.4 | 191.8 | 176.0 | 63.7 |
| 6.25 | 310.8 | 134.6 | 198.9 | 261.1 | 226.4 | 76.4 |
| 12.5 | 396.1 | 156.5 | 250.5 | 310.3 | 278.3 | 100.9 |
| 25 | 473.8 | 178.1 | 277.1 | 359.2 | 322.0 | 125.4 |
| 50 | 478.7 | 209.0 | 296.1 | 388.2 | 343.0 | 116.4 |
| 75 | 470.3 | 195.2 | 304.4 | 355.6 | 331.4 | 114.2 |
|  |  |  |  |  |  |  |
| POR-H2 | nmole Cytochrome C reduced/min/mg protein | | | | | |
| uM NADPH | Exp1 | Exp2 | Exp3 | Exp4 | Mean | SD |
| 0 | 0.0 | 0.0 | 0.0 | 0.0 | 0.0 | 0.0 |
| 1.5625 | 117.7 | 66.4 | 117.0 | 164.6 | 116.4 | 40.1 |
| 3.125 | 186.1 | 91.5 | 167.9 | 277.6 | 180.8 | 76.5 |
| 6.25 | 257.2 | 124.1 | 195.8 | 404.3 | 245.4 | 119.1 |
| 12.5 | 278.3 | 136.3 | 235.6 | 464.5 | 278.7 | 137.4 |
| 25 | 337.6 | 151.6 | 270.8 | 534.2 | 323.5 | 160.1 |
| 50 | 360.1 | 176.9 | 316.0 | 590.2 | 360.8 | 171.7 |
| 75 | 369.1 | 164.4 | 286.2 | 569.0 | 347.2 | 170.1 |
|  |  |  |  |  |  |  |
| POR-H3 | nmole Cytochrome C reduced/min/mg protein | | | | | |
| uM NADPH | Exp1 | Exp2 | Exp3 | Exp4 | Mean | SD |
| 0 | 0.0 | 0.0 | 0.0 | 0.0 | 0.0 | 0.0 |
| 1.5625 | 75.2 | 64.4 | 84.8 | 121.1 | 86.4 | 24.6 |
| 3.125 | 112.8 | 92.0 | 145.7 | 163.3 | 128.5 | 32.1 |
| 6.25 | 150.0 | 118.3 | 178.9 | 245.6 | 173.2 | 54.3 |
| 12.5 | 162.8 | 142.5 | 199.9 | 291.6 | 199.2 | 66.0 |
| 25 | 171.8 | 155.1 | 225.6 | 290.1 | 210.6 | 60.9 |
| 50 | 188.4 | 178.8 | 230.2 | 332.7 | 232.5 | 70.4 |
| 75 | 193.2 | 172.2 | 248.4 | 328.0 | 235.4 | 69.6 |
|  |  |  |  |  |  |  |
| POR-H4 | nmole Cytochrome C reduced/min/mg protein | | | | | |
| uM NADPH | Exp1 | Exp2 | Exp3 | Exp4 | Mean | SD |
| 0 | 0.0 | 0.0 | 0.0 | 0.0 | 0.0 | 0.0 |
| 1.5625 | 88.8 | 55.8 | 134.0 | 119.9 | 99.6 | 34.8 |
| 3.125 | 131.7 | 77.8 | 180.6 | 160.9 | 137.7 | 44.7 |
| 6.25 | 168.8 | 93.8 | 181.1 | 225.8 | 167.4 | 54.8 |
| 12.5 | 208.2 | 106.1 | 236.9 | 250.3 | 200.4 | 65.2 |
| 25 | 222.7 | 122.9 | 265.7 | 271.2 | 220.6 | 68.6 |
| 50 | 240.6 | 130.0 | 292.6 | 305.3 | 242.1 | 79.8 |
| 75 | 231.9 | 131.9 | 279.1 | 292.7 | 233.9 | 72.8 |

**S11 Table.**  Results of an enzyme kinetic study evaluating the effect of POR mutations on cytochrome c reduction by POR expressed in Sf9 cells. Shown are Michaelis-Menten parameters Km, Vmax and CLint (intrinsic clearance; Vmax / Km) for microsomal cytochrome c reduction by POR-H1 and each POR variant (H2-H4) when NADPH concentration is varied and cytochrome c concentration is fixed. Enzyme kinetic parameters were derived by fitting a one-enzyme Michaelis-Menten model to measured enzyme activities and substrate concentrations (data in Table S10) using nonlinear regression. Microsomal samples from each of four independently generated protein preparations (Experiment # 1-4) were independently tested three times each. The parameters shown below are the average of 3 independent incubations of each protein preparation. Other details are provided in the Materials and Methods section. SD – standard deviation.

|  | POR-H1 | | | | | |
| --- | --- | --- | --- | --- | --- | --- |
|  | Exp1 | Exp2 | Exp3 | Exp4 | Mean | SD |
| Km (uM) | 3.51 | 3.08 | 3.17 | 3.03 | 3.20 | 0.21 |
| Vmax (nmol/min/mg protein) | 510.50 | 206.20 | 313.60 | 391.74 | 355.51 | 128.30 |
| CLint (mL/min/mg protein) | 145.52 | 66.88 | 99.06 | 129.15 | 110.15 | 34.67 |
|  |  |  |  |  |  |  |
|  | POR-H2 | | | | | |
|  | Exp1 | Exp2 | Exp3 | Exp4 | Mean | SD |
| Km (uM) | 3.42 | 2.73 | 2.90 | 3.78 | 3.21 | 0.48 |
| Vmax (nmol/min/mg protein) | 381.59 | 174.10 | 306.70 | 615.95 | 369.59 | 185.30 |
| CLint (mL/min/mg protein) | 111.46 | 63.76 | 105.78 | 163.04 | 111.01 | 40.69 |
|  |  |  |  |  |  |  |
|  | POR-H3 | | | | | |
|  | Exp1 | Exp2 | Exp3 | Exp4 | Mean | SD |
| Km (uM) | 2.32 | 3.08 | 2.60 | 2.94 | 2.74 | 0.34 |
| Vmax (nmol/min/mg protein) | 195.79 | 180.59 | 249.07 | 344.10 | 242.39 | 73.89 |
| CLint (mL/min/mg protein) | 84.24 | 58.61 | 95.85 | 117.11 | 88.95 | 24.38 |
|  |  |  |  |  |  |  |
|  | POR-H4 | | | | | |
|  | Exp1 | Exp2 | Exp3 | Exp4 | Mean | SD |
| Km (uM) | 2.77 | 2.39 | 2.19 | 2.59 | 2.49 | 0.25 |
| Vmax (nmol/min/mg protein) | 247.92 | 133.82 | 287.56 | 307.80 | 244.27 | 77.72 |
| CLint (mL/min/mg protein) | 89.59 | 55.94 | 131.08 | 118.72 | 98.83 | 33.47 |

**S12 Table.**  Effect of POR mutations on cytochrome c reduction by POR co-expressed with (A) CYP2B11 or (B) CYP2D15 in Sf9 cells. Shown are rates of microsomal cytochrome c reduction by POR-H1 and each POR variant (H2-H4) at fixed NADPH concentration. Microsomal samples from each of four independently generated protein preparations (Experiment # 1-4) were independently tested three times each. The activities shown below are the average of 3 independent incubations of each protein preparation. Other details are provided in the Materials and Methods section. SD – standard deviation

|  | nmole Cytochrome C reduced/min/mg protein | | | | | |
| --- | --- | --- | --- | --- | --- | --- |
| CYP2B11 | Exp1 | Exp2 | Exp3 | Exp4 | Mean | SD |
| PFEV | 11.1 | 7.0 | 10.0 | 6.0 | 8.5 | 2.4 |
| POR-H1 | 183.9 | 250.0 | 115.1 | 204.8 | 188.5 | 56.1 |
| POR-H2 | 177.7 | 169.5 | 162.2 | 105.9 | 153.8 | 32.6 |
| POR-H3 | 142.8 | 91.1 | 150.2 | 144.8 | 132.2 | 27.6 |
| POR-H4 | 28.3 | 60.9 | 116.7 | 95.5 | 75.3 | 38.9 |
|  |  |  |  |  |  |  |
|  | nmole Cytochrome C reduced/min/mg protein | | | | | |
| CYP2D15 | Exp1 | Exp2 | Exp3 | Exp4 | Mean | SD |
| PFEV | 8.8 | 6.5 | 5.8 | 6.3 | 6.8 | 1.4 |
| POR-H1 | 133.4 | 161.9 | 154.7 | 229.1 | 169.8 | 41.3 |
| POR-H2 | 134.9 | 135.9 | 162.0 | 199.8 | 158.1 | 30.5 |
| POR-H3 | 50.1 | 150.8 | 142.5 | 116.7 | 115.0 | 45.6 |
| POR-H4 | 88.3 | 94.1 | 87.2 | 165.3 | 108.7 | 37.9 |

**S13 Table.**  Results of an enzyme kinetic study evaluating the effect of POR mutations on CYP2B11 enzyme function when coexpressed in Sf9 cells. Shown are rates of resorufin formation at different benzyloxyresorufin concentrations by CYP2B11 coexpressed with POR-H1, POR-H2, POR-H3, POR-H4, or pFastBac1 empty vector (PFEV) negative control in Sf9 microsomes. Microsomal samples from each of four independently generated protein preparations (Experiment # 1-4) were independently tested three times each. The activities shown below are the average of 3 independent incubations of each protein preparation. Other details are provided in the Materials and Methods section. SD – standard deviation

| PFEV | pmol resorufin/min/pmol P450 | | | | | |
| --- | --- | --- | --- | --- | --- | --- |
| uM BROD | Exp1 | Exp2 | Exp3 | Exp4 | Mean | SD |
| 0 | 0.000 | 0.000 | 0.000 | 0.000 | 0.000 | 0.000 |
| 0.5 | 0.015 | 0.013 | 0.035 | 0.015 | 0.020 | 0.011 |
| 0.75 | 0.020 | 0.018 | 0.047 | 0.025 | 0.027 | 0.013 |
| 1 | 0.028 | 0.028 | 0.047 | 0.028 | 0.033 | 0.010 |
| 2.5 | 0.050 | 0.032 | 0.099 | 0.047 | 0.057 | 0.029 |
| 5 | 0.047 | 0.047 | 0.099 | 0.037 | 0.057 | 0.028 |
| 10 | 0.053 | 0.062 | 0.121 | 0.070 | 0.076 | 0.031 |
|  |  |  |  |  |  |  |
| POR-H1 | pmol resorufin/min/pmol P450 | | | | | |
| uM BROD | Exp1 | Exp2 | Exp3 | Exp4 | Mean | SD |
| 0 | 0.000 | 0.000 | 0.000 | 0.000 | 0.000 | 0.000 |
| 0.5 | 0.893 | 0.776 | 0.879 | 0.508 | 0.764 | 0.178 |
| 0.75 | 1.328 | 1.246 | 1.454 | 0.802 | 1.208 | 0.283 |
| 1 | 1.670 | 1.615 | 1.754 | 1.066 | 1.526 | 0.312 |
| 2.5 | 2.375 | 2.267 | 2.431 | 1.440 | 2.128 | 0.464 |
| 5 | 2.622 | 2.437 | 2.663 | 1.564 | 2.322 | 0.515 |
| 10 | 2.985 | 3.057 | 2.945 | 1.727 | 2.679 | 0.636 |
|  |  |  |  |  |  |  |
| POR-H2 | pmol resorufin/min/pmol P450 | | | | | |
| uM BROD | Exp1 | Exp2 | Exp3 | Exp4 | Mean | SD |
| 0 | 0.000 | 0.000 | 0.000 | 0.000 | 0.000 | 0.000 |
| 0.5 | 0.851 | 0.664 | 0.678 | 0.585 | 0.694 | 0.112 |
| 0.75 | 1.298 | 1.060 | 1.090 | 0.865 | 1.078 | 0.177 |
| 1 | 1.653 | 1.369 | 1.278 | 1.134 | 1.358 | 0.219 |
| 2.5 | 2.275 | 1.884 | 1.705 | 1.484 | 1.837 | 0.334 |
| 5 | 2.367 | 2.116 | 1.839 | 1.671 | 1.998 | 0.307 |
| 10 | 3.490 | 2.254 | 1.984 | 2.039 | 2.442 | 0.709 |
|  |  |  |  |  |  |  |
| POR-H3 | pmol resorufin/min/pmol P450 | | | | | |
| uM BROD | Exp1 | Exp2 | Exp3 | Exp4 | Mean | SD |
| 0 | 0.000 | 0.000 | 0.000 | 0.000 | 0.000 | 0.000 |
| 0.5 | 0.752 | 0.453 | 0.412 | 0.357 | 0.494 | 0.177 |
| 0.75 | 1.157 | 0.741 | 0.607 | 0.549 | 0.764 | 0.274 |
| 1 | 1.439 | 0.861 | 0.723 | 0.715 | 0.935 | 0.343 |
| 2.5 | 1.971 | 1.278 | 0.952 | 1.010 | 1.303 | 0.467 |
| 5 | 2.042 | 1.388 | 1.083 | 1.061 | 1.393 | 0.457 |
| 10 | 2.196 | 1.603 | 1.379 | 1.178 | 1.589 | 0.440 |
|  |  |  |  |  |  |  |
| POR-H4 | pmol resorufin/min/pmol P450 | | | | | |
| uM BROD | Exp1 | Exp2 | Exp3 | Exp4 | Average | SD |
| 0 | 0.000 | 0.000 | 0.000 | 0.000 | 0.000 | 0.000 |
| 0.5 | 0.118 | 0.249 | 0.381 | 0.262 | 0.252 | 0.108 |
| 0.75 | 0.200 | 0.430 | 0.587 | 0.363 | 0.395 | 0.160 |
| 1 | 0.219 | 0.518 | 0.718 | 0.449 | 0.476 | 0.206 |
| 2.5 | 0.357 | 0.643 | 0.976 | 0.652 | 0.657 | 0.253 |
| 5 | 0.368 | 0.658 | 1.050 | 0.696 | 0.693 | 0.279 |
| 10 | 0.431 | 0.654 | 1.298 | 0.818 | 0.800 | 0.367 |

**S14 Table.**  Results of an enzyme kinetic study evaluating the effect of POR mutations on CYP2B11 enzyme function when coexpressed in Sf9 cells. Shown are rates of 4-hydroxypropofol formation at different propofol concentrations by CYP2B11 coexpressed with POR-H1, POR-H2, POR-H3, POR-H4, or pFastBac1 empty vector (PFEV) negative control in Sf9 microsomes. Microsomal samples from each of four independently generated protein preparations (Experiment # 1-4) were independently tested three times each. The activities shown below are the average of 3 independent incubations of each protein preparation. Other details are provided in the Materials and Methods section. SD – standard deviation

| PFEV | pmol 4OH-Propofol/min/pmol P450 | | | | | |
| --- | --- | --- | --- | --- | --- | --- |
| uM Propofol | Exp1 | Exp2 | Exp3 | Exp4 | Mean | SD |
| 0 | 0.00 | 0.00 | 0.00 | 0.00 | 0.00 | 0.00 |
| 2.5 | 0.00 | 0.00 | 0.00 | 0.00 | 0.00 | 0.00 |
| 5 | 0.00 | 0.18 | 0.16 | 0.17 | 0.13 | 0.08 |
| 10 | 0.10 | 0.38 | 0.27 | 0.32 | 0.27 | 0.12 |
| 25 | 0.18 | 0.49 | 0.76 | 0.63 | 0.52 | 0.25 |
| 50 | 0.35 | 0.62 | 0.88 | 0.75 | 0.65 | 0.23 |
| 75 | 0.41 | 0.66 | 0.96 | 0.81 | 0.71 | 0.24 |
| 100 | 0.44 | 0.81 | 0.93 | 0.87 | 0.76 | 0.22 |
|  |  |  |  |  |  |  |
| POR-H1 | pmol 4OH-Propofol/min/pmol P450 | | | | | |
| uM Propofol | Exp1 | Exp2 | Exp3 | Exp4 | Mean | SD |
| 0 | 0.00 | 0.00 | 0.00 | 0.00 | 0.00 | 0.00 |
| 2.5 | 4.05 | 4.55 | 5.47 | 3.66 | 4.44 | 0.78 |
| 5 | 5.93 | 5.97 | 7.13 | 4.38 | 5.85 | 1.13 |
| 10 | 7.25 | 7.63 | 8.55 | 5.39 | 7.21 | 1.33 |
| 25 | 10.03 | 9.25 | 11.44 | 6.49 | 9.31 | 2.08 |
| 50 | 12.18 | 11.52 | 14.27 | 7.54 | 11.38 | 2.82 |
| 75 | 13.39 | 12.27 | 16.97 | 8.81 | 12.86 | 3.36 |
| 100 | 15.06 | 13.33 | 15.52 | 9.82 | 13.43 | 2.59 |
|  |  |  |  |  |  |  |
| POR-H2 | pmol 4OH-Propofol/min/pmol P450 | | | | | |
| uM Propofol | Exp1 | Exp2 | Exp3 | Exp4 | Mean | SD |
| 0 | 0.00 | 0.00 | 0.00 | 0.00 | 0.00 | 0.00 |
| 2.5 | 3.24 | 4.19 | 3.77 | 3.32 | 3.63 | 0.44 |
| 5 | 5.64 | 5.47 | 5.14 | 4.11 | 5.09 | 0.69 |
| 10 | 6.95 | 7.73 | 7.00 | 5.63 | 6.83 | 0.87 |
| 25 | 9.77 | 9.64 | 7.97 | 6.23 | 8.40 | 1.66 |
| 50 | 11.31 | 11.36 | 10.14 | 8.20 | 10.25 | 1.48 |
| 75 | 13.50 | 12.92 | 11.21 | 8.81 | 11.61 | 2.11 |
| 100 | 13.24 | 12.98 | 11.67 | 9.34 | 11.81 | 1.78 |
|  |  |  |  |  |  |  |
| POR-H3 | pmol 4OH-Propofol/min/pmol P450 | | | | | |
| uM Propofol | Exp1 | Exp2 | Exp3 | Exp4 | Mean | SD |
| 0 | 0.00 | 0.00 | 0.00 | 0.00 | 0.00 | 0.00 |
| 2.5 | 3.21 | 2.99 | 1.63 | 2.08 | 2.48 | 0.75 |
| 5 | 4.68 | 4.02 | 2.30 | 2.68 | 3.42 | 1.12 |
| 10 | 6.50 | 5.34 | 3.13 | 3.51 | 4.62 | 1.58 |
| 25 | 8.51 | 6.76 | 3.91 | 4.65 | 5.96 | 2.09 |
| 50 | 9.99 | 8.03 | 4.79 | 4.91 | 6.93 | 2.53 |
| 75 | 11.36 | 8.64 | 5.15 | 5.39 | 7.64 | 2.95 |
| 100 | 11.95 | 9.14 | 5.40 | 6.38 | 8.22 | 2.95 |
|  |  |  |  |  |  |  |
| POR-H4 | pmol 4OH-Propofol/min/pmol P450 | | | | | |
| uM Propofol | Exp1 | Exp2 | Exp3 | Exp4 | Average | SD |
| 0 | 0.00 | 0.00 | 0.00 | 0.00 | 0.00 | 0.00 |
| 2.5 | 0.25 | 1.35 | 2.00 | 1.47 | 1.27 | 0.74 |
| 5 | 0.58 | 1.89 | 2.88 | 1.49 | 1.71 | 0.95 |
| 10 | 1.10 | 2.47 | 3.05 | 1.96 | 2.15 | 0.83 |
| 25 | 1.60 | 3.28 | 4.74 | 2.59 | 3.05 | 1.32 |
| 50 | 1.95 | 3.76 | 5.54 | 2.99 | 3.56 | 1.51 |
| 75 | 2.21 | 4.51 | 5.99 | 3.27 | 3.99 | 1.63 |
| 100 | 2.50 | 5.03 | 5.75 | 3.64 | 4.23 | 1.45 |

**S15 Table.**  Results of an enzyme kinetic study evaluating the effect of POR mutations on CYP2B11 enzyme function when coexpressed in Sf9 cells. Shown are rates of 6-hydroxybupropion formation at different bupropion concentrations by CYP2B11 coexpressed with POR-H1, POR-H2, POR-H3, POR-H4, or pFastBac1 empty vector (PFEV) negative control in Sf9 microsomes. Microsomal samples from each of four independently generated protein preparations (Experiment # 1-4) were independently tested three times each. The activities shown below are the average of 3 independent incubations of each protein preparation. Other details are provided in the Materials and Methods section. SD – standard deviation

| PFEV | pmol OH-Bupropion/min/pmol P450 | | | | | |
| --- | --- | --- | --- | --- | --- | --- |
| uM Bupropion | Exp1 | Exp2 | Exp3 | Exp4 | Mean | SD |
| 0 | 0.00 | 0.00 | 0.00 | 0.00 | 0.00 | 0.00 |
| 5 | 0.00 | 0.00 | 0.00 | 0.00 | 0.00 | 0.00 |
| 10 | 0.00 | 0.00 | 0.06 | 0.06 | 0.03 | 0.03 |
| 25 | 0.00 | 0.00 | 0.15 | 0.09 | 0.06 | 0.07 |
| 50 | 0.06 | 0.09 | 0.17 | 0.11 | 0.11 | 0.05 |
| 100 | 0.18 | 0.13 | 0.31 | 0.26 | 0.22 | 0.08 |
| 250 | 0.34 | 0.21 | 0.42 | 0.36 | 0.33 | 0.09 |
| 500 | 0.47 | 0.32 | 0.57 | 0.61 | 0.49 | 0.13 |
|  |  |  |  |  |  |  |
| POR-H1 | pmol OH-Bupropion/min/pmol P450 | | | | | |
| uM Bupropion | Exp1 | Exp2 | Exp3 | Exp4 | Average | SD |
| 0 | 0.00 | 0.00 | 0.00 | 0.00 | 0.00 | 0.00 |
| 5 | 0.96 | 0.69 | 0.82 | 0.85 | 0.83 | 0.11 |
| 10 | 1.05 | 0.95 | 1.11 | 1.15 | 1.06 | 0.09 |
| 25 | 1.54 | 1.32 | 1.80 | 1.50 | 1.54 | 0.20 |
| 50 | 1.88 | 1.48 | 2.14 | 1.84 | 1.83 | 0.27 |
| 100 | 2.57 | 2.22 | 2.83 | 2.54 | 2.54 | 0.25 |
| 250 | 3.08 | 2.73 | 3.75 | 3.24 | 3.20 | 0.42 |
| 500 | 3.75 | 3.10 | 4.49 | 3.86 | 3.80 | 0.57 |
|  |  |  |  |  |  |  |
| POR-H2 | pmol OH-Bupropion/min/pmol P450 | | | | | |
| uM Bupropion | Exp1 | Exp2 | Exp3 | Exp4 | Average | SD |
| 0 | 0.00 | 0.00 | 0.00 | 0.00 | 0.00 | 0.00 |
| 5 | 0.65 | 0.57 | 0.59 | 0.75 | 0.64 | 0.08 |
| 10 | 0.83 | 0.86 | 0.72 | 1.07 | 0.87 | 0.15 |
| 25 | 1.37 | 1.18 | 1.17 | 1.55 | 1.32 | 0.18 |
| 50 | 1.90 | 1.36 | 1.47 | 1.98 | 1.68 | 0.31 |
| 100 | 2.31 | 1.98 | 1.94 | 2.42 | 2.16 | 0.24 |
| 250 | 3.00 | 2.63 | 2.44 | 3.40 | 2.87 | 0.42 |
| 500 | 3.49 | 2.67 | 2.97 | 4.01 | 3.29 | 0.59 |
|  |  |  |  |  |  |  |
| POR-H3 | pmol OH-Bupropion/min/pmol P450 | | | | | |
| uM Bupropion | Exp1 | Exp2 | Exp3 | Exp4 | Average | SD |
| 0 | 0.00 | 0.00 | 0.00 | 0.00 | 0.00 | 0.00 |
| 5 | 0.64 | 0.44 | 0.28 | 0.40 | 0.44 | 0.15 |
| 10 | 0.89 | 0.58 | 0.43 | 0.69 | 0.65 | 0.19 |
| 25 | 1.25 | 0.83 | 0.66 | 0.91 | 0.91 | 0.25 |
| 50 | 1.74 | 1.16 | 0.88 | 1.16 | 1.24 | 0.36 |
| 100 | 2.18 | 1.40 | 0.99 | 1.46 | 1.51 | 0.49 |
| 250 | 2.77 | 1.76 | 1.36 | 2.05 | 1.99 | 0.59 |
| 500 | 3.17 | 2.02 | 1.50 | 2.39 | 2.27 | 0.70 |
|  |  |  |  |  |  |  |
| POR-H4 | pmol OH-Bupropion/min/pmol P450 | | | | | |
| uM Bupropion | Exp1 | Exp2 | Exp3 | Exp4 | Average | SD |
| 0 | 0.00 | 0.00 | 0.00 | 0.00 | 0.00 | 0.00 |
| 5 | 0.00 | 0.18 | 0.39 | 0.31 | 0.22 | 0.17 |
| 10 | 0.19 | 0.22 | 0.60 | 0.51 | 0.38 | 0.20 |
| 25 | 0.31 | 0.38 | 0.74 | 0.72 | 0.54 | 0.23 |
| 50 | 0.45 | 0.48 | 1.01 | 0.86 | 0.70 | 0.28 |
| 100 | 0.62 | 0.66 | 1.27 | 1.22 | 0.94 | 0.35 |
| 250 | 0.89 | 0.88 | 1.68 | 1.58 | 1.26 | 0.43 |
| 500 | 1.10 | 1.11 | 1.97 | 2.01 | 1.55 | 0.51 |

**S16 Table.**  Results of an enzyme kinetic study evaluating the effect of POR mutations on CYP2D15 enzyme function when coexpressed in Sf9 cells. Shown are rates of O-desmethyltramadol formation at different tramadol concentrations by CYP2D15 coexpressed with POR-H1, POR-H2, POR-H3, POR-H4, or pFastBac1 empty vector (PFEV) negative control in Sf9 microsomes. Microsomal samples from each of four independently generated protein preparations (Experiment # 1-4) were independently tested three times each. The activities shown below are the average of 3 independent incubations of each protein preparation. Other details are provided in the Materials and Methods section. SD – standard deviation

| PFEV | pmoles O-desmethyltramadol/min/pmol P450 | | | | | |
| --- | --- | --- | --- | --- | --- | --- |
| uM Tramadol | Exp1 | Exp2 | Exp3 | Exp4 | Mean | SD |
| 0 | 0.00 | 0.00 | 0.00 | 0.00 | 0.00 | 0.00 |
| 1 | 0.11 | 0.13 | 0.10 | 0.02 | 0.09 | 0.05 |
| 2 | 0.23 | 0.21 | 0.17 | 0.10 | 0.18 | 0.06 |
| 5 | 0.38 | 0.38 | 0.34 | 0.21 | 0.33 | 0.08 |
| 10 | 0.65 | 0.67 | 0.55 | 0.28 | 0.54 | 0.18 |
| 20 | 0.96 | 0.83 | 0.73 | 0.26 | 0.70 | 0.30 |
| 50 | 1.18 | 1.23 | 1.03 | 0.43 | 0.97 | 0.37 |
| 100 | 1.42 | 1.46 | 1.31 | 0.49 | 1.17 | 0.46 |
|  |  |  |  |  |  |  |
| POR-H1 | pmoles O-desmethyltramadol/min/pmol P450 | | | | | |
| uM Tramadol | Exp1 | Exp2 | Exp3 | Exp4 | Mean | SD |
| 0 | 0.00 | 0.00 | 0.00 | 0.00 | 0.00 | 0.00 |
| 1 | 1.27 | 0.85 | 1.95 | 1.90 | 1.49 | 0.53 |
| 2 | 2.05 | 1.47 | 3.19 | 2.85 | 2.39 | 0.78 |
| 5 | 4.08 | 2.43 | 6.05 | 5.03 | 4.40 | 1.53 |
| 10 | 5.93 | 4.16 | 8.35 | 6.61 | 6.26 | 1.73 |
| 20 | 7.76 | 5.16 | 10.55 | 5.92 | 7.35 | 2.40 |
| 50 | 9.75 | 6.53 | 13.10 | 10.39 | 9.94 | 2.70 |
| 100 | 10.29 | 6.99 | 13.98 | 11.44 | 10.67 | 2.90 |
|  |  |  |  |  |  |  |
| POR-H2 | pmoles O-desmethyltramadol/min/pmol P450 | | | | | |
| uM Tramadol | Exp1 | Exp2 | Exp3 | Exp4 | Mean | SD |
| 0 | 0.00 | 0.00 | 0.00 | 0.00 | 0.00 | 0.00 |
| 1 | 0.71 | 1.07 | 1.86 | 1.64 | 1.32 | 0.53 |
| 2 | 1.17 | 1.93 | 3.16 | 2.56 | 2.20 | 0.85 |
| 5 | 2.19 | 3.49 | 5.75 | 4.94 | 4.09 | 1.58 |
| 10 | 3.24 | 4.97 | 8.42 | 7.20 | 5.96 | 2.31 |
| 20 | 4.25 | 6.20 | 10.62 | 9.05 | 7.53 | 2.85 |
| 50 | 5.19 | 7.82 | 12.81 | 10.74 | 9.14 | 3.33 |
| 100 | 5.92 | 8.32 | 14.10 | 11.71 | 10.02 | 3.62 |
|  |  |  |  |  |  |  |
| POR-H3 | pmoles O-desmethyltramadol/min/pmol P450 | | | | | |
| uM Tramadol | Exp1 | Exp2 | Exp3 | Exp4 | Mean | SD |
| 0 | 0.00 | 0.00 | 0.00 | 0.00 | 0.00 | 0.00 |
| 1 | 0.58 | 1.59 | 1.58 | 1.16 | 1.23 | 0.48 |
| 2 | 0.56 | 2.62 | 2.93 | 1.93 | 2.01 | 1.05 |
| 5 | 1.69 | 4.69 | 5.49 | 3.37 | 3.81 | 1.66 |
| 10 | 2.41 | 6.19 | 7.05 | 4.43 | 5.02 | 2.05 |
| 20 | 3.03 | 7.66 | 8.22 | 5.48 | 6.10 | 2.36 |
| 50 | 3.80 | 8.73 | 10.05 | 6.59 | 7.29 | 2.73 |
| 100 | 4.46 | 9.28 | 10.41 | 6.88 | 7.76 | 2.64 |
|  |  |  |  |  |  |  |
| POR-H4 | pmoles O-desmethyltramadol/min/pmol P450 | | | | | |
| uM Tramadol | Exp1 | Exp2 | Exp3 | Exp4 | Mean | SD |
| 0 | 0.00 | 0.00 | 0.00 | 0.00 | 0.00 | 0.00 |
| 1 | 1.45 | 1.49 | 1.94 | 1.47 | 1.59 | 0.24 |
| 2 | 1.28 | 2.50 | 3.29 | 2.33 | 2.35 | 0.83 |
| 5 | 3.99 | 4.56 | 5.90 | 3.80 | 4.56 | 0.95 |
| 10 | 6.04 | 6.41 | 8.21 | 5.18 | 6.46 | 1.28 |
| 20 | 7.25 | 8.13 | 10.33 | 5.33 | 7.76 | 2.08 |
| 50 | 8.56 | 9.84 | 12.10 | 6.77 | 9.32 | 2.24 |
| 100 | 9.02 | 10.34 | 13.15 | 7.19 | 9.93 | 2.51 |

**S17 Table.**  Results of an enzyme kinetic study evaluating the effect of POR mutations on CYP2D15 enzyme function when coexpressed in Sf9 cells. Shown are rates of dextrorphan formation at different dextromethorphan concentrations by CYP2D15 coexpressed with POR-H1, POR-H2, POR-H3, POR-H4, or pFastBac1 empty vector (PFEV) negative control in Sf9 microsomes. Microsomal samples from each of four independently generated protein preparations (Experiment # 1-4) were independently tested three times each. The activities shown below are the average of 3 independent incubations of each protein preparation. Other details are provided in the Materials and Methods section. SD – standard deviation

| PFEV | pmol dextrorphan formed/min/pmol P450 | | | | | |
| --- | --- | --- | --- | --- | --- | --- |
| uM Dextromethorphan | Exp1 | Exp2 | Exp3 | Exp4 | Mean | SD |
| 0 | 0.00 | 0.00 | 0.00 | 0.00 | 0.00 | 0.00 |
| 0.1 | 0.00 | 0.00 | 0.04 | 0.03 | 0.02 | 0.02 |
| 0.25 | 0.12 | 0.16 | 0.13 | 0.06 | 0.12 | 0.04 |
| 0.5 | 0.29 | 0.37 | 0.26 | 0.16 | 0.27 | 0.08 |
| 1 | 0.51 | 0.86 | 0.45 | 0.34 | 0.54 | 0.22 |
| 5 | 0.90 | 1.38 | 0.84 | 0.59 | 0.93 | 0.33 |
| 10 | 1.03 | 1.67 | 0.95 | 0.68 | 1.08 | 0.42 |
| 25 | 1.18 | 1.70 | 1.17 | 0.71 | 1.19 | 0.40 |
|  |  |  |  |  |  |  |
| POR-H1 | pmol dextrorphan formed/min/pmol P450 | | | | | |
| uM Dextromethorphan | Exp1 | Exp2 | Exp3 | Exp4 | Mean | SD |
| 0 | 0.00 | 0.00 | 0.00 | 0.00 | 0.00 | 0.00 |
| 0.1 | 0.44 | 0.42 | 0.97 | 0.71 | 0.64 | 0.26 |
| 0.25 | 1.26 | 1.20 | 2.11 | 1.64 | 1.55 | 0.42 |
| 0.5 | 2.20 | 1.86 | 3.23 | 2.57 | 2.46 | 0.58 |
| 1 | 3.58 | 2.91 | 4.83 | 3.85 | 3.79 | 0.80 |
| 5 | 4.73 | 4.06 | 6.63 | 5.19 | 5.15 | 1.09 |
| 10 | 5.45 | 4.41 | 6.96 | 5.68 | 5.62 | 1.05 |
| 25 | 5.48 | 4.74 | 7.41 | 5.98 | 5.90 | 1.13 |
|  |  |  |  |  |  |  |
| POR-H2 | pmol dextrorphan formed/min/pmol P450 | | | | | |
| uM Dextromethorphan | Exp1 | Exp2 | Exp3 | Exp4 | Mean | SD |
| 0 | 0.00 | 0.00 | 0.00 | 0.00 | 0.00 | 0.00 |
| 0.1 | 0.28 | 0.56 | 0.86 | 0.67 | 0.59 | 0.24 |
| 0.25 | 0.78 | 1.35 | 2.00 | 1.80 | 1.48 | 0.54 |
| 0.5 | 1.09 | 2.21 | 3.10 | 2.56 | 2.24 | 0.85 |
| 1 | 2.05 | 3.39 | 4.40 | 4.16 | 3.50 | 1.06 |
| 5 | 2.76 | 4.44 | 6.60 | 5.31 | 4.78 | 1.61 |
| 10 | 2.91 | 4.81 | 6.94 | 5.61 | 5.06 | 1.69 |
| 25 | 3.03 | 4.65 | 7.34 | 6.21 | 5.31 | 1.88 |
|  |  |  |  |  |  |  |
| POR-H3 | pmol dextrorphan formed/min/pmol P450 | | | | | |
| uM Dextromethorphan | Exp1 | Exp2 | Exp3 | Exp4 | Mean | SD |
| 0 | 0.00 | 0.00 | 0.00 | 0.00 | 0.00 | 0.00 |
| 0.1 | 0.25 | 0.69 | 0.66 | 0.38 | 0.49 | 0.21 |
| 0.25 | 0.64 | 1.49 | 1.33 | 1.18 | 1.16 | 0.37 |
| 0.5 | 0.97 | 2.40 | 2.41 | 1.73 | 1.88 | 0.69 |
| 1 | 1.58 | 4.25 | 3.61 | 3.03 | 3.12 | 1.14 |
| 5 | 2.23 | 6.16 | 4.81 | 3.90 | 4.27 | 1.65 |
| 10 | 2.23 | 6.37 | 5.13 | 4.00 | 4.43 | 1.76 |
| 25 | 2.46 | 6.68 | 5.68 | 4.24 | 4.76 | 1.83 |
|  |  |  |  |  |  |  |
| POR-H4 | pmol dextrorphan formed/min/pmol P450 | | | | | |
| uM Dextromethorphan | Exp1 | Exp2 | Exp3 | Exp4 | Mean | SD |
| 0 | 0.00 | 0.00 | 0.00 | 0.00 | 0.00 | 0.00 |
| 0.1 | 0.59 | 0.75 | 1.04 | 0.59 | 0.74 | 0.21 |
| 0.25 | 1.63 | 1.68 | 2.13 | 1.18 | 1.66 | 0.39 |
| 0.5 | 2.38 | 2.75 | 2.91 | 1.83 | 2.47 | 0.48 |
| 1 | 3.67 | 4.52 | 4.97 | 2.97 | 4.03 | 0.89 |
| 5 | 4.70 | 5.95 | 6.34 | 3.87 | 5.22 | 1.14 |
| 10 | 5.55 | 6.20 | 7.23 | 4.68 | 5.92 | 1.08 |
| 25 | 5.91 | 7.01 | 7.70 | 4.92 | 6.39 | 1.22 |

**S18 Table.**  Results of an enzyme kinetic study evaluating the effect of POR mutations on CYP2B11 enzyme function when coexpressed in Sf9 cells. Shown are Michaelis-Menten parameters Km, Vmax and CLint (intrinsic clearance; Vmax / Km) for microsomal resorufin formation at different benzyloxyresorufin concentrations by CYP2B11 coexpressed with POR-H1, POR-H2, POR-H3, POR-H4, or pFastBac1 empty vector (PFEV) negative control in Sf9 microsomes. Enzyme kinetic parameters were derived by fitting a one-enzyme Michaelis-Menten model to measured enzyme activities and substrate concentrations using nonlinear regression. Microsomal samples from each of four independently generated protein preparations (Experiment # 1-4) were independently tested three times each. The parameters shown below are the average of 3 independent incubations of each protein preparation. Other details are provided in the Materials and Methods section. SD – standard deviation.

|  | PFEV | | | | | |
| --- | --- | --- | --- | --- | --- | --- |
|  | Exp1 | Exp2 | Exp3 | Exp4 | Mean | SD |
| Km (uM) | 1.21 | 2.30 | 1.49 | 1.64 | 1.66 | 0.46 |
| Vmax (pmol/min/pmol P450) | 0.06 | 0.07 | 0.14 | 0.07 | 0.09 | 0.04 |
| Clint (µL/min/pmol P450) | 0.05 | 0.03 | 0.09 | 0.04 | 0.05 | 0.03 |
|  |  |  |  |  |  |  |
|  | POR-H1 | | | | | |
|  | Exp1 | Exp2 | Exp3 | Exp4 | Mean | SD |
| Km (uM) | 1.10 | 1.27 | 0.99 | 1.01 | 1.09 | 0.13 |
| Vmax (pmol/min/pmol P450) | 3.30 | 3.32 | 3.26 | 1.92 | 2.95 | 0.69 |
| Clint (µL/min/pmol P450) | 2.99 | 2.61 | 3.30 | 1.91 | 2.70 | 0.60 |
|  |  |  |  |  |  |  |
|  | POR-H2 | | | | | |
|  | Exp1 | Exp2 | Exp3 | Exp4 | Mean | SD |
| Km (uM) | 1.48 | 1.03 | 0.81 | 1.15 | 1.12 | 0.28 |
| Vmax (pmol/min/pmol P450) | 3.65 | 2.55 | 2.17 | 2.19 | 2.64 | 0.69 |
| Clint (µL/min/pmol P450) | 2.46 | 2.47 | 2.68 | 1.90 | 2.38 | 0.34 |
|  |  |  |  |  |  |  |
|  | POR-H3 | | | | | |
|  | Exp1 | Exp2 | Exp3 | Exp4 | Mean | SD |
| Km (uM) | 0.82 | 1.13 | 1.14 | 0.99 | 1.02 | 0.15 |
| Vmax (pmol/min/pmol P450) | 2.44 | 1.77 | 1.44 | 1.31 | 1.74 | 0.50 |
| Clint (µL/min/pmol P450) | 2.96 | 1.57 | 1.26 | 1.32 | 1.78 | 0.80 |
|  |  |  |  |  |  |  |
|  | POR-H4 | | | | | |
|  | Exp1 | Exp2 | Exp3 | Exp4 | Mean | SD |
| Km (uM) | 1.17 | 0.59 | 1.07 | 1.07 | 0.98 | 0.26 |
| Vmax (pmol/min/pmol P450) | 0.48 | 0.74 | 1.38 | 0.89 | 0.87 | 0.38 |
| Clint (µL/min/pmol P450) | 0.41 | 1.25 | 1.28 | 0.83 | 0.94 | 0.41 |

**S19 Table.**  Results of an enzyme kinetic study evaluating the effect of POR mutations on CYP2B11 enzyme function when coexpressed in Sf9 cells. Shown are Michaelis-Menten parameters Km, Vmax and CLint (intrinsic clearance; Vmax / Km) for microsomal 4-hydroxypropofol formation at different propofol concentrations by CYP2B11 coexpressed with POR-H1, POR-H2, POR-H3, POR-H4, or pFastBac1 empty vector (PFEV) negative control in Sf9 microsomes. Enzyme kinetic parameters were derived by fitting a one-enzyme Michaelis-Menten model to measured enzyme activities and substrate concentrations using nonlinear regression. Microsomal samples from each of four independently generated protein preparations (Experiment # 1-4) were independently tested three times each. The parameters shown below are the average of 3 independent incubations of each protein preparation. Other details are provided in the Materials and Methods section. SD – standard deviation.

|  | PFEV | | | | | |
| --- | --- | --- | --- | --- | --- | --- |
|  | Exp1 | Exp2 | Exp3 | Exp4 | Mean | SD |
| Km (uM) | 21.18 | 20.01 | 25.68 | 23.40 | 22.57 | 2.51 |
| Vmax (pmol/min/pmol P450) | 0.58 | 0.90 | 1.27 | 1.09 | 0.96 | 0.30 |
| Clint (µL/min/pmol P450) | 0.03 | 0.05 | 0.05 | 0.05 | 0.04 | 0.01 |
|  |  |  |  |  |  |  |
|  | POR-H1 | | | | | |
|  | Exp1 | Exp2 | Exp3 | Exp4 | Mean | SD |
| Km (uM) | 9.58 | 6.55 | 7.93 | 5.90 | 7.49 | 1.63 |
| Vmax (pmol/min/pmol P450) | 15.20 | 13.22 | 16.94 | 9.21 | 13.64 | 3.32 |
| Clint (µL/min/pmol P450) | 1.59 | 2.02 | 2.14 | 1.56 | 1.83 | 0.30 |
|  |  |  |  |  |  |  |
|  | POR-H2 | | | | | |
|  | Exp1 | Exp2 | Exp3 | Exp4 | Mean | SD |
| Km (uM) | 9.53 | 7.36 | 6.91 | 6.43 | 7.56 | 1.37 |
| Vmax (pmol/min/pmol P450) | 14.30 | 13.54 | 11.81 | 9.31 | 12.24 | 2.21 |
| Clint (µL/min/pmol P450) | 1.50 | 1.84 | 1.71 | 1.45 | 1.62 | 0.18 |
|  |  |  |  |  |  |  |
|  | POR-H3 | | | | | |
|  | Exp1 | Exp2 | Exp3 | Exp4 | Mean | SD |
| Km (uM) | 8.91 | 6.84 | 7.56 | 6.39 | 7.43 | 1.10 |
| Vmax (pmol/min/pmol P450) | 12.40 | 9.30 | 5.58 | 6.04 | 8.33 | 3.18 |
| Clint (µL/min/pmol P450) | 1.39 | 1.36 | 0.74 | 0.94 | 1.11 | 0.32 |
|  |  |  |  |  |  |  |
|  | POR-H4 | | | | | |
|  | Exp1 | Exp2 | Exp3 | Exp4 | Mean | SD |
| Km (uM) | 19.02 | 9.69 | 7.09 | 8.74 | 11.14 | 5.37 |
| Vmax (pmol/min/pmol P450) | 2.84 | 5.00 | 6.25 | 4.37 | 4.62 | 1.42 |
| Clint (µL/min/pmol P450) | 0.15 | 0.52 | 0.88 | 0.50 | 0.51 | 0.30 |

**S20 Table.**  Results of an enzyme kinetic study evaluating the effect of POR mutations on CYP2B11 enzyme function when coexpressed in Sf9 cells. Shown are Michaelis-Menten parameters Km, Vmax and CLint (intrinsic clearance; Vmax / Km) for microsomal 6-hydroxybupropion formation at different bupropion concentrations by CYP2B11 coexpressed with POR-H1, POR-H2, POR-H3, POR-H4, or pFastBac1 empty vector (PFEV) negative control in Sf9 microsomes. Enzyme kinetic parameters were derived by fitting a one-enzyme Michaelis-Menten model to measured enzyme activities and substrate concentrations using nonlinear regression. Microsomal samples from each of four independently generated protein preparations (Experiment # 1-4) were independently tested three times each. The parameters shown below are the average of 3 independent incubations of each protein preparation. Other details are provided in the Materials and Methods section. SD – standard deviation.

|  | PFEV | | | | | |
| --- | --- | --- | --- | --- | --- | --- |
|  | Exp1 | Exp2 | Exp3 | Exp4 | Mean | SD |
| Km (uM) | 488.31 | 357.33 | 131.57 | 383.42 | 340.16 | 150.14 |
| Vmax (pmol/min/pmol P450) | 0.95 | 0.54 | 0.69 | 1.04 | 0.80 | 0.23 |
| Clint (µL/min/pmol P450) | 0.00 | 0.00 | 0.01 | 0.00 | 0.00 | 0.00 |
|  |  |  |  |  |  |  |
|  | POR-H1 | | | | | |
|  | Exp1 | Exp2 | Exp3 | Exp4 | Mean | SD |
| Km (uM) | 34.71 | 36.92 | 45.87 | 40.52 | 39.50 | 4.87 |
| Vmax (pmol/min/pmol P450) | 3.67 | 3.15 | 4.57 | 3.87 | 3.82 | 0.59 |
| Clint (µL/min/pmol P450) | 0.11 | 0.09 | 0.10 | 0.10 | 0.10 | 0.01 |
|  |  |  |  |  |  |  |
|  | POR-H2 | | | | | |
|  | Exp1 | Exp2 | Exp3 | Exp4 | Mean | SD |
| Km (uM) | 41.54 | 36.68 | 43.05 | 45.94 | 41.80 | 3.87 |
| Vmax (pmol/min/pmol P450) | 3.58 | 2.85 | 3.00 | 4.08 | 3.38 | 0.57 |
| Clint (µL/min/pmol P450) | 0.09 | 0.08 | 0.07 | 0.09 | 0.08 | 0.01 |
|  |  |  |  |  |  |  |
|  | POR-H3 | | | | | |
|  | Exp1 | Exp2 | Exp3 | Exp4 | Mean | SD |
| Km (uM) | 37.58 | 33.27 | 33.79 | 46.83 | 37.87 | 6.28 |
| Vmax (pmol/min/pmol P450) | 3.23 | 2.02 | 1.52 | 2.45 | 2.31 | 0.72 |
| Clint (µL/min/pmol P450) | 0.09 | 0.06 | 0.05 | 0.05 | 0.06 | 0.02 |
|  |  |  |  |  |  |  |
|  | POR-H4 | | | | | |
|  | Exp1 | Exp2 | Exp3 | Exp4 | Mean | SD |
| Km (uM) | 92.04 | 63.45 | 40.51 | 56.56 | 63.14 | 21.53 |
| Vmax (pmol/min/pmol P450) | 1.26 | 1.17 | 1.99 | 2.07 | 1.62 | 0.47 |
| Clint (µL/min/pmol P450) | 0.01 | 0.02 | 0.05 | 0.04 | 0.03 | 0.02 |

**S21 Table.**  Results of an enzyme kinetic study evaluating the effect of POR mutations on CYP2D15 enzyme function when coexpressed in Sf9 cells. Shown are Michaelis-Menten parameters Km, Vmax and CLint (intrinsic clearance; Vmax / Km) for microsomal O-desmethyltramadol formation at different tramadol concentrations by CYP2D15 coexpressed with POR-H1, POR-H2, POR-H3, POR-H4, or pFastBac1 empty vector (PFEV) negative control in Sf9 microsomes. Enzyme kinetic parameters were derived by fitting a one-enzyme Michaelis-Menten model to measured enzyme activities and substrate concentrations using nonlinear regression. Microsomal samples from each of four independently generated protein preparations (Experiment # 1-4) were independently tested three times each. The parameters shown below are the average of 3 independent incubations of each protein preparation. Other details are provided in the Materials and Methods section. SD – standard deviation.

|  | PFEV | | | | | |
| --- | --- | --- | --- | --- | --- | --- |
|  | Exp1 | Exp2 | Exp3 | Exp4 | Mean | SD |
| Km (uM) | 14.32 | 17.08 | 18.73 | 9.99 | 15.03 | 3.82 |
| Vmax (pmol/min/pmol P450) | 1.59 | 1.67 | 1.50 | 0.51 | 1.32 | 0.54 |
| Clint (µL/min/pmol P450) | 0.11 | 0.10 | 0.08 | 0.05 | 0.09 | 0.03 |
|  |  |  |  |  |  |  |
|  | POR-H1 | | | | | |
|  | Exp1 | Exp2 | Exp3 | Exp4 | Mean | SD |
| Km (uM) | 8.89 | 9.26 | 7.65 | 8.21 | 8.50 | 0.72 |
| Vmax (pmol/min/pmol P450) | 11.30 | 7.67 | 14.96 | 11.58 | 11.38 | 2.98 |
| Clint (µL/min/pmol P450) | 1.27 | 0.83 | 1.96 | 1.41 | 1.37 | 0.47 |
|  |  |  |  |  |  |  |
|  | POR-H2 | | | | | |
|  | Exp1 | Exp2 | Exp3 | Exp4 | Mean | SD |
| Km (uM) | 9.48 | 7.93 | 7.89 | 7.51 | 8.20 | 0.87 |
| Vmax (pmol/min/pmol P450) | 6.33 | 8.95 | 15.00 | 12.48 | 10.69 | 3.82 |
| Clint (µL/min/pmol P450) | 0.67 | 1.13 | 1.90 | 1.66 | 1.34 | 0.55 |
|  |  |  |  |  |  |  |
|  | POR-H3 | | | | | |
|  | Exp1 | Exp2 | Exp3 | Exp4 | Mean | SD |
| Km (uM) | 10.37 | 5.44 | 5.46 | 5.90 | 6.79 | 2.39 |
| Vmax (pmol/min/pmol P450) | 4.76 | 9.72 | 10.94 | 7.26 | 8.17 | 2.74 |
| Clint (µL/min/pmol P450) | 0.46 | 1.79 | 2.00 | 1.23 | 1.37 | 0.69 |
|  |  |  |  |  |  |  |
|  | POR-H4 | | | | | |
|  | Exp1 | Exp2 | Exp3 | Exp4 | Mean | SD |
| Km (uM) | 7.43 | 7.13 | 6.72 | 4.49 | 6.44 | 1.33 |
| Vmax (pmol/min/pmol P450) | 9.85 | 11.11 | 13.86 | 7.26 | 10.52 | 2.75 |
| Clint (µL/min/pmol P450) | 1.33 | 1.56 | 2.06 | 1.62 | 1.64 | 0.31 |

**Table S22.**  Results of an enzyme kinetic study evaluating the effect of POR mutations on CYP2D15 enzyme function when coexpressed in Sf9 cells. Shown are Michaelis-Menten parameters Km, Vmax and CLint (intrinsic clearance; Vmax / Km) for microsomal dextrorphan formation at different dextromethorphan concentrations by CYP2D15 coexpressed with POR-H1, POR-H2, POR-H3, POR-H4, or pFastBac1 empty vector (PFEV) negative control in Sf9 microsomes. Enzyme kinetic parameters were derived by fitting a one-enzyme Michaelis-Menten model to measured enzyme activities and substrate concentrations using nonlinear regression. Microsomal samples from each of four independently generated protein preparations (Experiment # 1-4) were independently tested three times each. The parameters shown below are the average of 3 independent incubations of each protein preparation. Other details are provided in the Materials and Methods section. SD – standard deviation.

|  | PFEV | | | | | |
| --- | --- | --- | --- | --- | --- | --- |
|  | Exp1 | Exp2 | Exp3 | Exp4 | Mean | SD |
| Km (uM) | 1.71 | 1.57 | 1.93 | 1.93 | 1.79 | 0.18 |
| Vmax (pmol/min/pmol P450) | 1.24 | 1.86 | 1.20 | 1.20 | 1.37 | 0.32 |
| Clint (µL/min/pmol P450) | 0.72 | 1.18 | 0.62 | 0.62 | 0.79 | 0.27 |
|  |  |  |  |  |  |  |
|  | POR-H1 | | | | | |
|  | Exp1 | Exp2 | Exp3 | Exp4 | Mean | SD |
| Km (uM) | 0.76 | 0.75 | 0.63 | 0.65 | 0.70 | 0.07 |
| Vmax (pmol/min/pmol P450) | 5.70 | 4.79 | 7.51 | 6.05 | 6.01 | 1.13 |
| Clint (µL/min/pmol P450) | 7.50 | 6.39 | 12.01 | 9.26 | 8.79 | 2.45 |
|  |  |  |  |  |  |  |
|  | POR-H2 | | | | | |
|  | Exp1 | Exp2 | Exp3 | Exp4 | Mean | SD |
| Km (uM) | 0.74 | 0.59 | 0.71 | 0.62 | 0.66 | 0.07 |
| Vmax (pmol/min/pmol P450) | 3.15 | 4.96 | 7.51 | 6.15 | 5.44 | 1.85 |
| Clint (µL/min/pmol P450) | 4.25 | 8.42 | 10.58 | 9.96 | 8.30 | 2.85 |
|  |  |  |  |  |  |  |
|  | POR-H3 | | | | | |
|  | Exp1 | Exp2 | Exp3 | Exp4 | Mean | SD |
| Km (uM) | 0.70 | 0.81 | 0.68 | 0.64 | 0.71 | 0.07 |
| Vmax (pmol/min/pmol P450) | 2.50 | 6.99 | 5.64 | 4.36 | 4.87 | 1.91 |
| Clint (µL/min/pmol P450) | 3.54 | 8.65 | 8.32 | 6.85 | 6.84 | 2.33 |
|  |  |  |  |  |  |  |
|  | POR-H4 | | | | | |
|  | Exp1 | Exp2 | Exp3 | Exp4 | Mean | SD |
| Km (uM) | 0.70 | 0.70 | 0.68 | 0.79 | 0.72 | 0.05 |
| Vmax (pmol/min/pmol P450) | 5.86 | 6.94 | 7.69 | 4.93 | 6.35 | 1.21 |
| Clint (µL/min/pmol P450) | 8.41 | 9.95 | 11.24 | 6.27 | 8.97 | 2.14 |
